# Spatiotemporal Molecular Architecture of Lineage Allocation and Cellular Organization in Tooth Morphogenesis

**DOI:** 10.1002/advs.202403627

**Published:** 2024-11-13

**Authors:** Shengjie Jiang, Yuning Zhang, Huimin Zheng, Kai Zhao, Yue Yang, Binbin Lai, Xuliang Deng, Yan Wei

**Affiliations:** ^1^ Department of Geriatric Dentistry Beijing Laboratory of Biomedical Materials Peking University School and Hospital of Stomatology Beijing 100081 P. R. China; ^2^ Institute of Medical Technology Peking University Health Science Center Beijing 100191 P. R. China; ^3^ Department of Orthodontics Peking University School and Hospital of Stomatology Beijing 100081 P. R. China; ^4^ Department of Prosthodontics The First Clinical Division Peking University School and Hospital of Stomatology Beijing 100081 P. R. China; ^5^ Biomedical Engineering Department Institute of Advanced Clinical Medicine Peking University Beijing 100191 P. R. China; ^6^ Department of Dermatology Peking University First Hospital Beijing 100034 P. R. China

**Keywords:** spatial transcriptome, single‐cell RNA sequencing, tooth morphogenesis

## Abstract

The remarkable evolution of teeth morphological complexity represents a giant leap for vertebrate. Despite its importance in life history, the understanding of spatiotemporal organization of teeth remains rudimentary. Herein, a high‐resolution genome‐wide molecular patterning of lineage allocation and cellular organization in tooth morphogenesis is described, constructed by integrating spatial transcriptome and single‐cell RNA sequencing. Twelve spatial compartments and seventeen heterogeneous cell clusters linked to tooth morphogenic milestones are identified. Eighty‐eight percent of total lineage species has already appeared in the initial tooth bud rather than the generally considered sequential emergence. A previously unrecognized sprouting‐like patterning mode of the dental papilla is discovered, that the inner compartment can break through the outer shell compartment to build up the final papilla cusp. Meanwhile, the continuum differentiation hierarchies of enamel knots in time and space are revealed. Furthermore, the regulatory network directing tooth morphogenesis is established, whereby a series of mechanotransduction signals are spatiotemporally involved beyond the well‐established classical odontogenesis signals. Finally, genes underlying tooth dysplasia are successfully tracked to highly specific time points and cell types. The results raise the idea that tooth morphogenesis is orchestrated by mechanical niches combined with biochemical signaling.

## Graphical abstract

It is found that tooth morphogenesis is regulated by a combination of mechanical niches and biochemical signaling, as revealed by a comprehensive map of tooth development using high‐throughput spatial transcriptome and single‐cell RNA sequencing. These findings establish a fundamental basis for understanding these essential elements, which could ultimately enhance regenerative techniques for specific tooth structures.

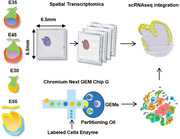

## 1 Introduction

Tooth morphogenesis shares many similarities with other embryonic organs, involving a series of sequential and reciprocal epithelial‐mesenchymal interactions. As such, teeth have long served as a model organ to study fundamental questions of developmental biology, including the determination, differentiation, and organization of tissues. The subtle heterotopic shifts in tooth morphology might play a large role in evolutionary transformation, which significantly increases the precision and efficiency of dental performance.^[^ ^1^ ^]^ Primates of the sophisticated morphology patterning in mammalian teeth might help to trace the strategy of life evolution and body plan.^[^ ^2^ ^]^

A global characterization of tooth morphogenesis across organogenesis has long been pursued. Histological analysis revealed that tooth enamel is derived from the oral ectoderm‐originated epithelium, while the remaining tooth structures originated from the cranial neural crest‐derived mesenchyme.^[^ ^3^ ^]^ Genetic profiling established the indispensable role of four conserved signaling pathways of transforming growth factor‐β (TGFβ), fibroblast growth factor (FGF), Sonic Hedgehog (SHH), and Wingless/Integrated (WNT) involved in tooth morphogenesis. Niche ablating studies further advanced the definition of odontogenesis at the molecular level.^[^ ^4^ ^]^ However, in these studies, the molecular make‐up of heterogeneous cell clusters and their localized distribution was confounded by the absence of high‐resolution spatial and temporal information. These limitations have resulted in controversies concerning the developmental trajectories and functional role of distinct cell types, and their localization during development.

Herein, In this study, we created a comprehensive map of tooth development using high‐throughput spatial transcriptome (ST) and single‐cell RNA sequencing (scRNA‐seq).^[^ ^5^ ^]^ We observed a sprouting‐like patterning mode in the dental papilla during tooth morphogenesis and proposed that the enamel knot was an intermediate stage of the IEE‐OEE cluster. Our results showed that mechanotransduction signals (MAPK, Hippo, PI3K‐AKT, and mTOR) play a role in regulating tooth morphogenesis. Based on our findings, we identified key cells and determinants linked to tooth dysplasia over time and space. Understanding these essential elements could improve regenerative techniques for specific tooth structures.

## 2 Results

### 2.1 Generating the Spatiotemporal Atlas for Tooth Morphogenesis

**Figure 1:**
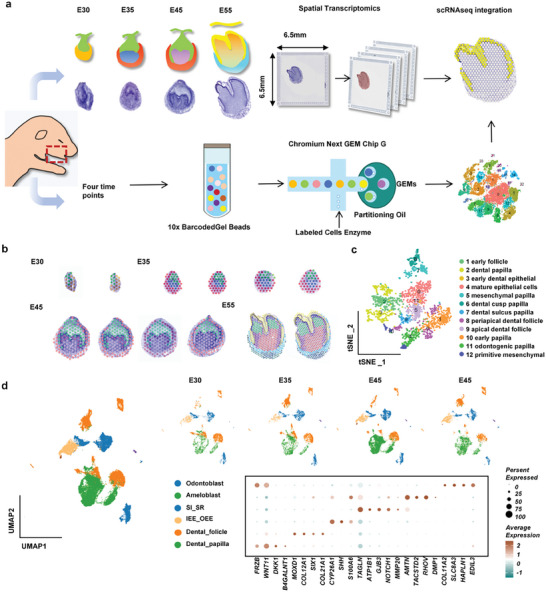
Generation of a spatiotemporal transcriptional atlas of tooth development. a) Overview of the molecular approach for the tooth development atlas. Exploring transcriptome‐wide spatiotemporal patterns in the morphological stages of tooth development by spatial transcriptome analysis (upper), and dissecting cell type heterogeneity of tooth germ at intermediate time point by scRNA‐seq (below). b,c) Spatial transcriptomic analysis of tooth germ samples from four developmental stages identifies twelve clusters of spots as viewed in four distinct histological regions of imaging slices (panel b) and a T‐SNE plot of integrated transcriptomic spots (panel c). d) Six major cell types were identified from integrated scRNA‐seq datasets from four teeth (E30–E35) germ developmental stages. UMAP plots of integrated (left panel) and separate samples (right upper) from four stages were shown. The dot plot shows the expression patterns of marker genes for each cluster (right bottom).

To clarify the cell heterogeneity and establish the spatiotemporal atlas for tooth morphogenesis, we investigated the developmental characteristics of miniature pig deciduous molar teeth germs using combined high‐throughput ST and scRNA‐seq (**Figure** **1**a). Our data on teeth germs ranged from developmental stages E35–E55, covering the four major developmental stages before tooth eruption: the bud stage, cap stage, bell stage, and crown stage.^[^ ^6^ ^]^ As a result, 12 spatially heterogeneous compartments were identified based on spatial transcriptome data, which linked to different anatomical regions of tooth germs when mapped back to the original coordinates in tissue sections (Figure 1b,c; Figure S1, Supporting Information).

Meanwhile, analysis on scRNA‐seq discovered 6 main cell groups (Figure 1d) which were further separated into 17 subclusters with specific highly‐expressed genes, indicating their specialized biological functions for tooth development (Figure S2, Supporting Information). The distribution of cell clusters during development showed that progenitor cell clusters tended to appear at earlier stages, while differentiation clusters appeared at later stages (Figure S3a, Supporting Information). Additionally, we delineated their relationships using partition‐based graph abstraction (Figure S3b, Supporting Information). Overall, our ST and scRNA‐seq data provide a comprehensive spatiotemporal transcriptomic profiling of tooth morphogenesis.

### 2.2 Epithelial and Mesenchymal Developmental Trajectories

**Figure 2:**
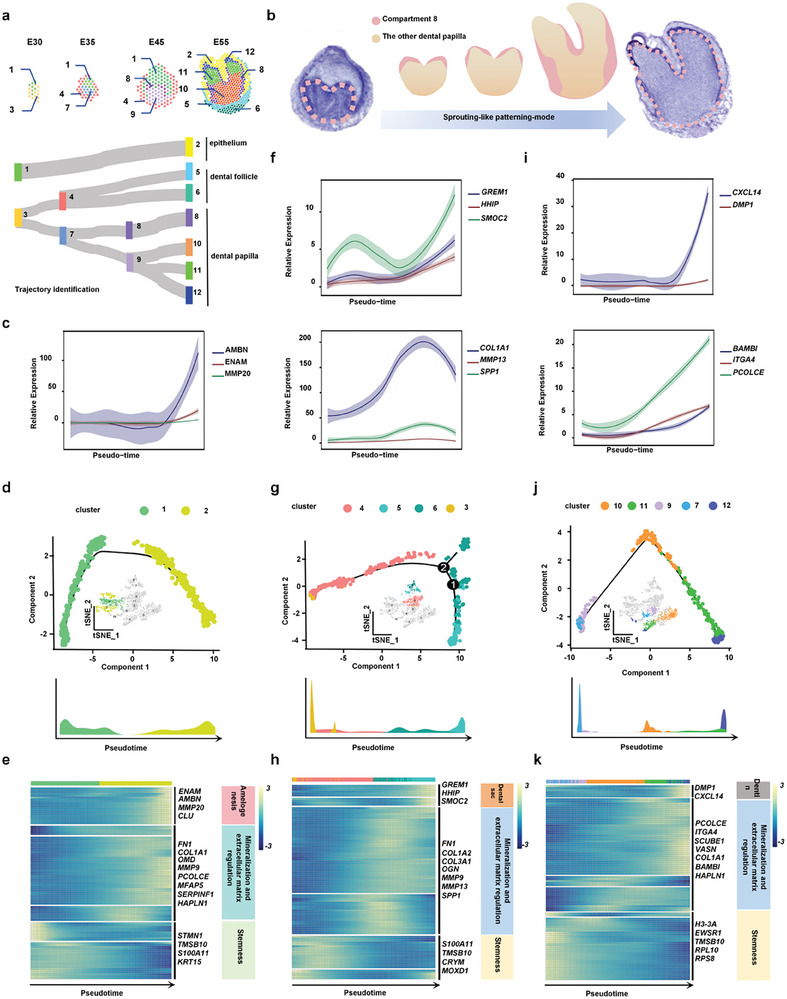
Phylogenetic trajectory analysis for different tooth developmental lineages. a) The developmental connections of tooth germ spatial domains based on differentiation trajectory analysis in tissues from E30‐E55 stages. b) A sprouting‐like patterning mode of cell compartments occurs when entering the crown stage. c) Dynamics of genes involved in dental epithelial development along the pseudotime trajectory in (d). d) Monocle lineage inference model representing the pseudotime trajectory of spatial spots in epithelium domains. e) Pseudotime heatmap representing the expression of the amelogenesis, matrix mineralization, and stemness marker genes ordered along the axis of the epithelium specification branch in (d). f) Dynamics of genes involved in dental follicle development along the pseudotime trajectory in (g). g) Monocle lineage inference model representing pseudotime trajectory of spatial spots in dental follicle domains. h) Pseudotime heatmap representing the expression of the dental follicle formation, matrix mineralization, and stemness marker genes ordered along the axis of follicle specification branch in (g). i) Dynamics of genes involved in dental papilla development along the pseudotime trajectory in (j). j) Monocle lineage inference model representing the pseudotime trajectory of spatial spots in dental papilla domains. k) Pseudotime heatmap representing the expression of the dentin formation, matrix mineralization, and stemness marker genes ordered along the axis of the papilla specification branch in (j).

To determine the developmental trajectories of the dental epithelium and mesenchyme, we undertook pseudo‐time analysis on the ST data and characterized gene expression changes along the trajectories. We found that the epithelium undertook a straightforward differentiation from early dental epithelial cells (compartment 1) into mature epithelial cells (compartment 2) with enamel formation function (**Figure** **2**a,d), while the mesenchyme has a more complex pattern of differentiation originated from the primitive mesenchyme (compartment 3) in the bud stage. The primitive papilla (compartment 7) and follicle (compartment 4) at the cap stage were separated from the primitive mesenchyme, which suggested that tooth germ heterogeneity was established at an early stage (Figure 2a). At the bell stage, the primitive papilla (compartment 7) differentiated into two downstream lineages in the apical area (compartment 8) and the coronal area (compartment 9), separately. Then, a sprouting‐like patterning of cell compartments occurred when entering the crown stage (Figure 2b). Compartment 9 further differentiated into a group of functionally graded cells (compartment 10, 11, and 12) that sprouted through compartment 8 and occupied the cusp area (Figure 2a,b,j), while compartment 8 migrated to the sulcus and sidewall of the papilla, rather than forming the dental cusp at the last stage. Meanwhile, compartment 4 of the dental mesenchyme follicle differentiated into compartments 5 and 6, which approached the apical foramina and wrapped the entire dental germ (Figure 2a,g).

We then examined the expression of key genes during the pseudo‐time of epithelial and mesenchymal development and found that genes related to mineralization and matrix modulation increased with epithelial and mesenchymal maturation, whereas stemness genes decreased significantly with dental germ maturation. (Figure 2e,h,k). Specifically, amelogenesis‐related genes *ENAM* and *AMBN* were upregulated along the epithelial pseudo‐time (Figure 2c; Figure S4, Supporting Information); odontogenesis‐related genes *DMP1* and *CXCL14* were increased along the dental papilla pseudo‐time; and periodontium‐related genes *GERM1* and *SMOC2* were upregulated along the dental follicle pseudo‐time (Figure 2f,i).

### 2.3 Molecular Biological Function Analysis Revealed the Epithelial‐Mesenchymal Spatial Functional Patterns

**Figure 3:**
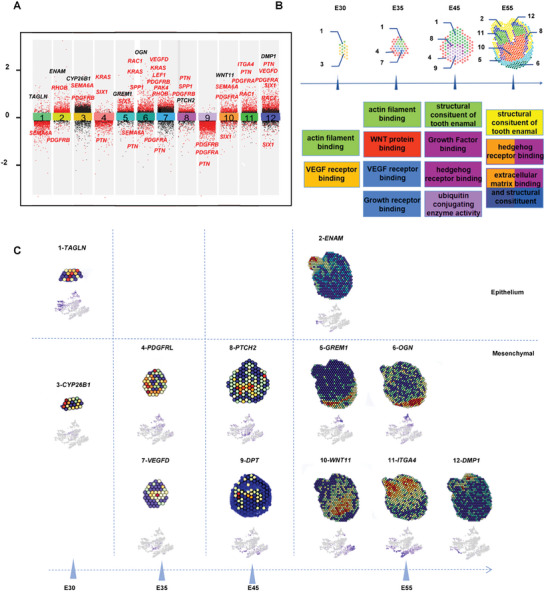
Molecular functions of specific spatial clusters for tooth morphogenesis. a) Differential gene expression analysis showing upregulated and downregulated genes from all twelve spatial clusters. Mechanically linked genes are highlighted in red. An adjusted *p*‐value < 0.01 is indicated in red, while an adjusted *p*‐value ≥ 0.01 is indicated in black. b) GO functions of twelve spatial clusters along the developmental stages showing biological signal transmission patterns for tooth morphogenesis. c) Spatial transcriptome sequencing slides illustrating the expression of function‐specific marker genes for the dental epithelium, follicle, and papilla. A color gradient of blue to red represents low to high expression levels.

To identify the spatial compartment function in tooth organ formation during embryonic development, we performed differential gene expression (DGE) analysis to determine the genes whose expression varied dramatically across the 12 compartments (**Figure** **3**a). Interestingly, we found that the differentially expressed genes among all the compartments contained mechanical‐related genes indicating that mechanical‐related signals play a pivotal role throughout all stages of tooth morphogenesis, underscoring the significance of mechanical cues in shaping dental development.^[^ ^7^ ^]^ We performed Gene ontology (GO) analysis on the DEGs across the 12 compartments. GO analysis showed that the primitive epithelial compartments were enriched with actin filament binding functions, suggesting their strong migration capability, from the bud stage to the cap stage (Figure 3b). Primitive epithelial compartment 1 existed from the bud stage to the bell stage, showing high expression of the cytoskeleton binding protein‐related gene *TAGLN*, implying their strong migration capability (Figure 3c). At crown stage, the epithelia enriched with structural constitution functions started to form tooth enamel (Figure 3b).

At the bud stage, the mesenchymal compartment 3 was enriched in VEGF receptor binding and suggested relatively fast proliferation with the upregulated expression of the embryo development‐related gene *CYP26B1* (Figure 3b,c). After entering the cap and bell stages, the mesenchyme was enriched in regulatory signaling of WNT, Hedgehog, and ubiquitination. At the bell stage, compartment 9 was located at the coronal location and exhibited specific expression of the cell‐matrix interaction and matrix assembly‐related gene DPT, indicating its ability to generate primary dentin tissue. The high expression of the SHH family receptor gene PTCH2 in compartment 8 indicated that it may be led to differentiate by SHH signals (Figure 3b,c).

Additionally, the structural constitution function was also observed in the mesenchyme at the crown stage, suggesting the construction of tooth dentin (Figure 3b). We discovered that compartment 12 near the tip of the tooth cusp displayed strong expression of the odontogenesis‐related gene *DMP1*, indicating the formation of mature odontoblasts at the crown stage. Below compartment 12, compartment 11 showed high expression of matrix recognition‐related gene *ITGA4*, indicating that it was involved in the formation of the cusp extracellular matrix (ECM). In compartment 10, located at the dental pulp position in the crown stage, upregulated *WNT11* indicated its differentiation potential (Figure 3c). Taken together, these results suggested that the dental epithelium had a strong migration capability, while the dental mesenchyme underwent abundant proliferation and differentiation behavior during tooth morphogenesis.

### 2.4 Establishment of Epithelial Cells Clusters

**Figure 4:**
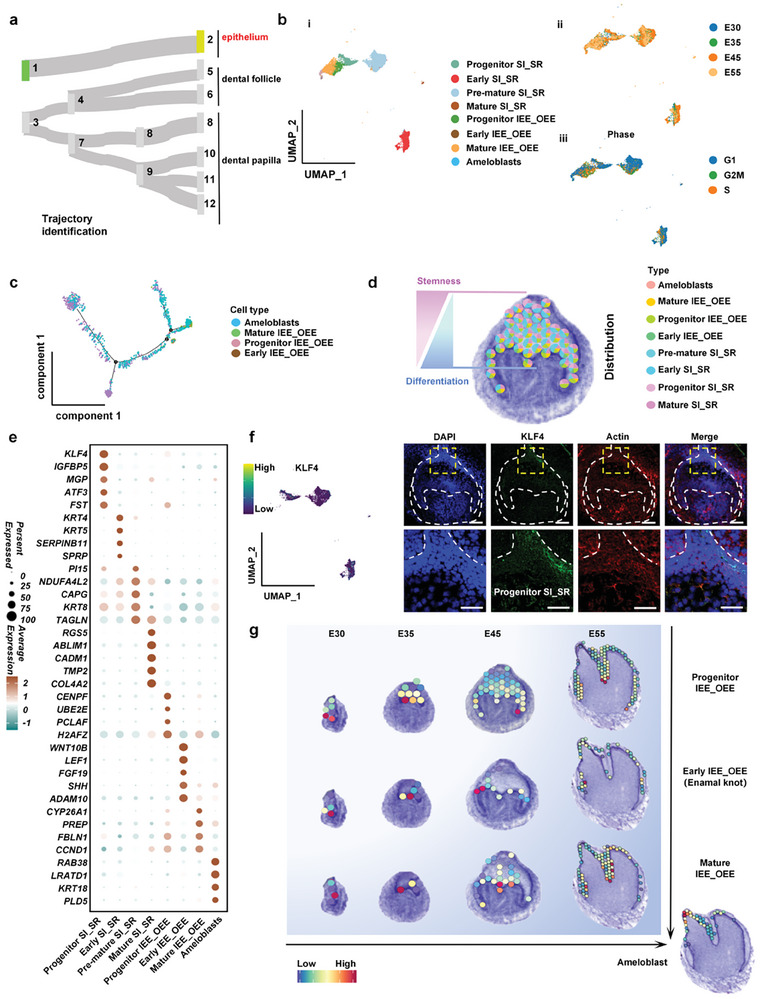
Cataloging in dental epithelial development. a) The developmental connections of dental epithelium domains. b) UMAP plot visualizing epithelial cell clusters (i) and epithelial cell distribution based on the developmental time course (ii) and cell cycle stage (iii). c) Pseudotemporal cell ordering visualizes the developmental course of IEE_OEE cells to ameloblasts. d) Conjoint analysis of single cell and spatial transcriptome sequencing representing the spatial distribution characteristics of epithelial clusters from the progenitor basal to differentiated interface. e) Dot plot of epithelial cell cluster markers. Points are scaled by the percentage of cells expressing the gene within the cluster and colored by the average expression level. f) UMAP plot and immunofluorescence staining representing *KLF4*+ progenitor SI_SR cells mainly distributed at the top of the tooth germ epithelium. Scale bars, 100 µm (up), 50 µm (down). g) Conjoint analysis of single cell and spatial transcriptome sequencing showing the spatial distributions of dental epithelial IEE_OEE clusters and Ameloblast at the bud stage, cap stage, bell stage, and differentiation stage.

After determining the compartments in the ST, we further matched the cell types derived from scRNA‐seq and the compartments from ST data. We first examined the developmental trajectory of epithelia across four stages (**Figure** **4**a). Based on single‐cell data, we identified eight epithelial cell clusters, including progenitor stratum intermedium_stellate reticulum (SI_SR) cells, early SI_SR cells, pre‐mature SI_SR cells, mature SI_SR cells, progenitor inner enamel epithelium_outer enamel epithelium (IEE_OEE) cells, early IEE_OEE cells, mature IEE_OEE cells, and ameloblasts (Figure 4bi). We found that the progenitor epithelial cells were mainly distributed at the bud stage and the differentiation clusters were distributed more at the bell stage and the crown stage (Figure 4bii; Figure S5, Supporting Information). Using cell cycle analysis, we found that the progenitor SI_SR and progenitor IEE_OEE had more G2/M phase cells^[^ ^8^ ^]^ implying the tendency to differentiate into mature clusters (Figure 4Biii). The differentiation trajectory from progenitor IEE_OEE cells to ameloblasts was corroborated using Pseudo‐time analysis (Figure 4c). Interestingly, we found that the percentage of progenitor cell clusters was gradually reduced from the coronal area of the dental epithelium to the epithelium‐mesenchyme interface, while the percentage of differentiated cell clusters was gradually increased (Figure 4d).

At the bud stage, we found that except for ameloblasts, all kinds of epithelial cell clusters had already appeared. Nevertheless, the main components of the epithelium at this stage were the progenitor SI_SR cells, mainly distributed at the coronal region, and the progenitor IEE_OEE cells, which were widely distributed at the epithelial‐mesenchymal interface (Figure 4d,g; Figure S6, Supporting Information). Differential gene expression analysis revealed that progenitor SI_SR cells had high expression of primitive markers (*KLF4*) and regulatory epithelial growth factor genes (*ATF3*, *MGP*, and *FST*) (Figure 4e,f). We investigated the function of this signaling center and discovered that it is associated with growth, morphology, and other factors, thereby corroborating our initial hypothesis (Figure S7, Supporting Information). The noval characterized *KLF4*+ progenitor SI_SR cell location was confirmed by immunofluorescence staining (Figure 4f). It might act as a signaling hub to modulate mesenchymal transcription and sustain pluripotency during tooth morphogenesis.^[^ ^9^ ^]^ In progenitor IEE_OEE cells, the cell division and proliferation regulating marker (*CENPF)* and a variety of proliferation‐related signals (*UBE2S*, *H2AFZ*, and *PCLAF)* were highly expressed, suggesting their capability to regulate the proliferation and differentiation of the dental mesenchyme (Figure 4e).

At the cap stage, progenitor SI_SR cells differentiated into three types of SI_SR clusters. Early SI_SR cells were localized near the tip of the epithelium and showed high expression of keratinization‐related markers (*KRT4* and *KRT5)*, revealing their supportive role in tooth morphogenesis.^[^ ^10^ ^]^ The pre‐mature SI_SR cells were widely distributed in the whole epithelial region, showing high expression of *KRT8* and *TAGLN*, indicating their importance in providing the contractile force during epithelial morphological maintenance. The mature SI_SR cells were localized closer to the epithelial‐mesenchymal interface and might act as the main source of epithelial support via their distinct expression of the actin filaments marker (*ABLIM1*) and the cell recognition marker (*CADM1*) (Figure 4e). Meanwhile, progenitor IEE_OEE cells also differentiated into two types of clusters: early IEE_ OEE cells, which were mainly distributed at the tip of future cusps, and mature IEE_OEE cells, which were mainly distributed in the future fossa, sulcus, and ridge (Figure 4g). We found that enamel knot markers (*WNT10B*, *FGF19*, and *SHH*) were highly expressed in early IEE_OEE clusters, suggesting this key inductive role in tooth morphogenesis.^[^ ^11^ ^]^ We found strong expression of FBLN1, which is critical for some developmental processes and contributes to the supramolecular organization of ECM architecture, particularly those of basement membranes, in mature IEE_OEE cells, suggesting the high mechanical property. Proliferation‐related gene *CCND1* was also found in mature IEE_OEE cells, which could accelerate condensation and crowding of interface cells, further promoting the migration of the epithelium (Figure 4f).

Entering the crown stage, the ST results revealed the first appearance of mature ameloblasts (Figure 4g). As the mature functional epithelial cells, ameloblasts specifically expressed enamel formation related *ENAM* (Figure S5b, Supporting Information). In addition, the spatial distributions of key epithelial clusters such as progenitor SI_SR cells, early SI_SR cells, mature SI_SR cells shown by single‐cell RNA‐seq analysis were verified by immunofluorescence staining (Figures S8 and S9, Supporting Information).

### 2.5 Dental Papilla Development

**Figure 5:**
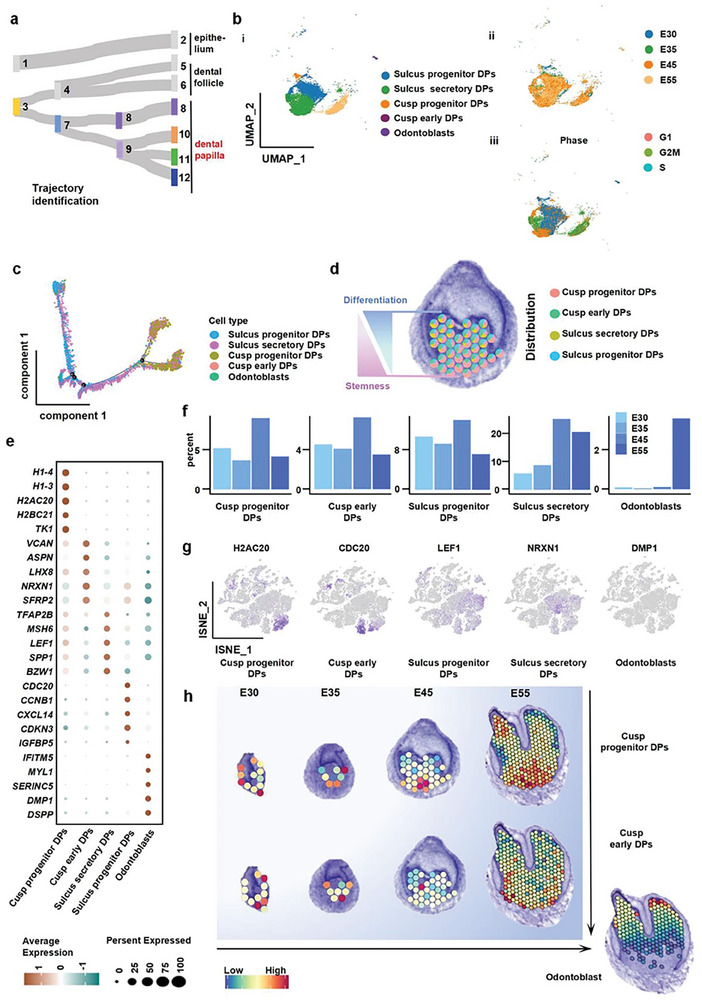
Spatiotemporal analysis of dental papilla development with spatial transcriptome sequencing and scRNA‐seq. a) The developmental connections of dental papilla domains. b) UMAP plot visualizing dental papilla cell clusters (i) and papilla cell distribution based on the developmental time course (ii) and cell cycle stage (iii). c) Pseudotemporal cell ordering visualizes the developmental course of dental papilla clusters. d) Conjoint analysis of single cell and spatial transcriptome sequencing representing the spatial distribution characteristics of the dental papilla clusters. e) Dot plot representing markers of specific cell clusters in the dental papilla compartment. Points are scaled by the percentage of cells expressing the gene within the cluster and colored by the average expression level. f) Bar plots representing the time distribution characteristics of dental papilla clusters, indicating the occurrence sequence of progenitor clusters and differentiated clusters. g) A T‐SNE plot showed the marker genes of five dental papilla clusters. h) Conjoint analysis of single cell and spatial transcriptome sequencing showing the spatial distributions of cusp progenitor DPs, cusp early DPs, and Odontoblast at the bud stage, cap stage, bell stage, and the differentiation stage.

The hard dentin tissue and the soft pulp tissue develop from the dental papilla (DP). To clarify the development trajectory of the DP in tooth morphogenesis, we depicted their gene expression and spatial distribution characteristics. The DP cells initially developed from the primitive mesenchyme and gradually differentiated into four clusters with different functions (**Figure** **5**a). From scRNA‐seq data, we identified five clusters covering different stages of DP differentiation. Pulp and cusp progenitor DP cells, cusp early DP cells, Sulcus secretory DP cells appeared at the bud stage (Figure 5b(i)). Meanwhile, matured odontoblasts were identified at the crown stage (Figure 5b(ii),f,h). Cell cycle analysis was conducted to clarify the potential of the progenitor cell clusters to differentiate into mature cell clusters (Figure 5b(iii)). Pseudo‐time analysis further depicted the differentiation trajectory from progenitor cell clusters to mature cell clusters (Figure 5c). By combining scRNA‐seq and ST data, we found that the stemness cell clusters were mainly localized at the bottom of the proliferative papilla compartment, while differentiated cell clusters gradually migrated to the epithelium‐mesenchyme interface (Figure 5h; Figure S10, Supporting Information).

At the bud stage, we found that except for matured odontoblasts, all types of papilla clusters appeared and two progenitor cell clusters accounted for the highest proportion (Figure 5f). In the early stage of development, cusp progenitor DP cells exhibited many primitive features, including high expression of *TK1*, which is important to regulate chromosome structure, *H2AC20*, which is involved in gene transcription, and *H1‐3*, which is related to cell division (Figure 5e,f). Another progenitor clusters, the sulcus progenitor DP cells, expressed cell cycle regulation genes (*CDC20*, *CCNB1*, and *CDKN3*), suggesting the role of cell proliferation and condensation in cusp formation (Figure 5e,g).

During the cap stage, the proportion of cusp progenitor DP cells and sulcus progenitor DP cells decreased as the differentiated cell clusters increased. The sulcus secretory DP cells were located at compartment 8 at the bell stage with high expression of tooth growth regulatory factors (*TFAP2B*, *LEF1*, and *MSH6*), which induced the development and differentiation of the dental papilla (Figure 5d; Figure S10a, Supporting Information). *SSP1* was also highly expressed in sulcus secretory DP cells, suggesting it might lead to an increase in the stiffness of the extracellular matrix in this region. This might further affect the migration direction of the dental epithelium and the spatial position of the tooth cusp at the bell stage. Cusp early DP cells showed high expression of cell mineralization genes (*VCAN* and *ASPN*), neurogenesis markers (*LHX8* and *NRXN1*), and angiogenesis markers (*SFRP2*), which further contributed to the formation of the cusp and complex pulp tissue. Finally, at the crown stage, matured odontoblasts were formed at the epithelium‐mesenchyme interface. The odontoblasts distinctly expressed dentin markers (*DMP1* and *DSPP*) and extracellular matrix secretion regulation genes (*IFITM5*, *MYL1*, and *SERINC5*), allowing them to secrete dentin in the future (Figure 5e). After entering the crown stage, matured odontoblasts sprouted out to the cusp area of the papilla after sulcus secretory DP cells migrated to the side wall and sulcus of the tooth. Cusp progenitors DP cells and cusp early DP cells continued to move downward and were primarily distributed near the future apical foramina (Figure 5h). We utilized the cell2location method to transcribe the single‐celled transcriptome data of pig tooth germs for joint analysis of data and spatial distribution, as depicted in the figure. The figure illustrates the distribution of mature_IEE_OEE, Sulcus_secretory DPs, and Pre_mature_SI_SR cells during the E45 period. The results were consistent with the characteristics of tooth germ development (Figure S11). Taken together, we recapitulated the cascade process of fate transition of dental papilla mesenchymal cells from rapid proliferation to differentiation.

### 2.6 Dental Follicle Developed During Periodontal Tissue Development

**Figure 6:**
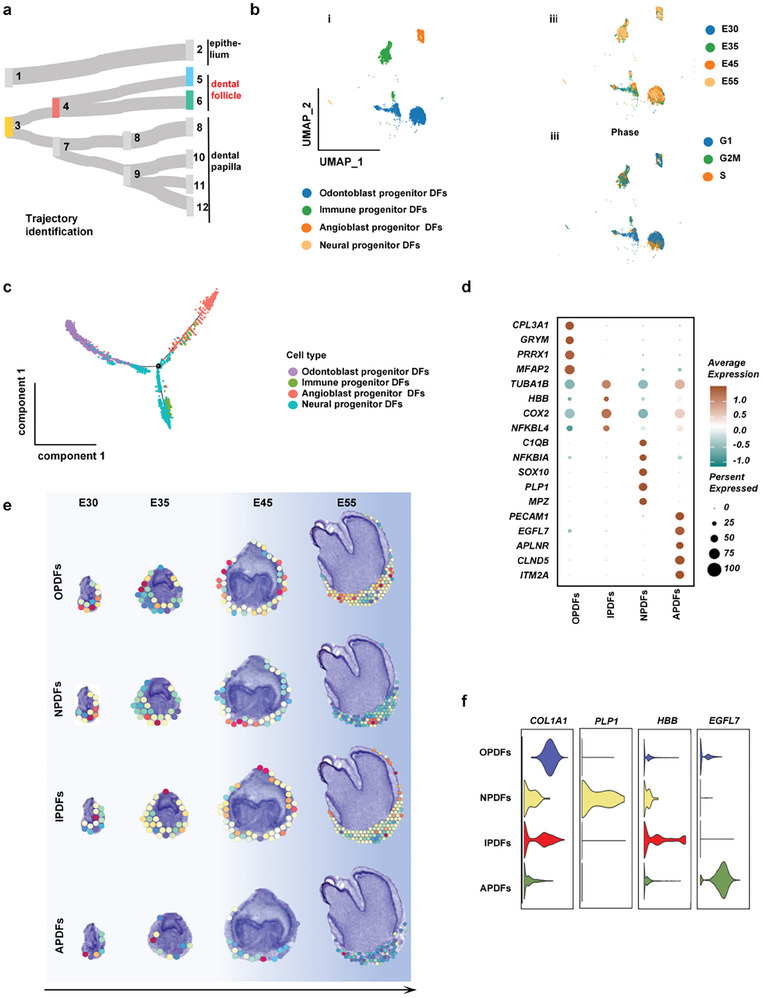
Cataloging in dental follicle development. a) The developmental connections of dental follicle domains. b) UMAP plot visualizing dental follicle cell clusters (i) and the follicle cell distribution based on the developmental time course (ii) and cell cycle stage (iii). c) Pseudotemporal cell ordering visualizes the developmental course of dental follicle clusters. d) Dot plot representing markers of specific dental follicle clusters. Points were scaled by the percentage of cells expressing the gene within the clusters, colored by average expression level. e) Conjoint analysis of single cell and spatial transcriptome sequencing representing the spatial distribution characteristics of dental follicle clusters. f) Violin plots presenting the expression level of selected markers closely related to osteogenesis, neurogenesis, angiogenesis, and immunogenicity functions. h) UMAP plot representing four spatial clusters of follicle clusters based on functional cluster status.

Next, we examined dental mesenchymal follicle development (**Figure** **6**a), which is essential for the periodontal tissue formation. From single‐cell RNA‐seq data, besides the major cell cluster of odontoblast progenitor denticle follicle (DF) cells, previously unrecognized immune progenitor DF cells, angioblast progenitor DF cells, and neural progenitors DF cells were also detected in the dental mesenchymal follicle (Figure 6b(i); Figure S12a, Supporting Information). Cell cycle analysis clarified that odontoblast progenitor clusters had the potential for rapid proliferation (Figure 6b(ii)). Time distribution and cell proportion analysis also showed that the proportion of dental follicle progenitor clusters was high in the early period and gradually reduced from the bud stage to the crown stage (Figure 6b(iii),c; Figure S12b, Supporting Information). High expression of extracellular matrix‐related genes (*COL3A1*, *CRYM*, and *MFAP4*) for odontoblast progenitor DF cells might be related to their function of the generation of alveolar bone and the periodontium, while high expression of immune regulatory genes (*HBB* and *NFKBIA*) for immune progenitor DF cells would account for immune adjustment during periodontal development (Figure 6d). Both odontoblast progenitor DF cells and immune progenitor DF cells were widely distributed in the dental follicle, indicating the critical role of immunoregulation in periodontal bone tissue development and (Figure 6e; Figure S12c, Supporting Information). Additionally, neural progenitor DF cells and angioblast progenitor DF cells were also found in the dental follicle, which would be responsible for neovascularization of periodontal tissue. With enhanced expression of nerve‐related genes (*MPZ*, *PLP1*, and *SOX10*), neural progenitor DF cells were localized at the pulp perforation area, which would generate periodontal nerves connecting with the dental pulp. With high expression of endothelial‐related genes (*EGFL7*, *PECAM1*, and *ITM2A*), angioblast progenitor DF cells showed a dispersed distribution in the dental follicle where they would engineer periodontal blood vessels (Figure 6e,f).

### 2.7 The Cell‐Cell Communication Analysis Reveals Spatiotemporal Epithelial‐Mesenchyme Interaction

**Figure 7:**
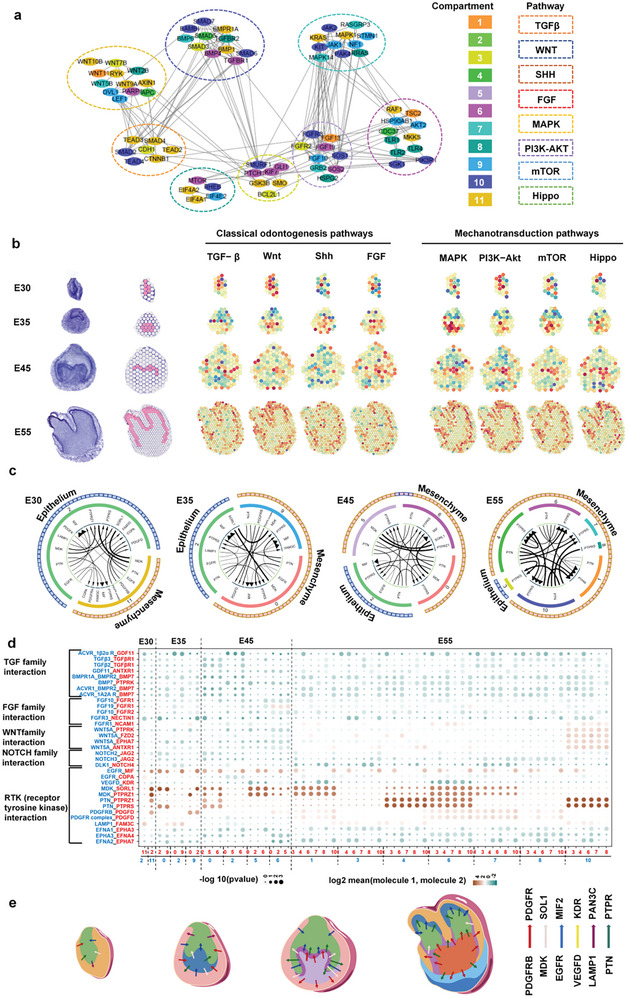
The regulatory network directing spatiotemporal epithelia‐mesenchyme interaction. a) Graph representation of the morphogen molecule STRING interactome. Pathways are shown by dashed ellipses. Genes from various pathways were linked if they were co‐expressed. The compartments where genes are highly expressed are labeled in different colors. b) Conjoint analysis of KEGG pathways and spatial transcriptome sequencing showing the dental‐specific morphogen spatial module at four stages. c) Circos plots showing representative ligand‐receptor interactions between epithelial clusters and mesenchymal clusters at four stages. d) Summary of the selected ligand‐receptor interactions among different spatial clusters. P‐values (permutation test) are displayed by the size of the circle. Interaction levels are displayed by color gradient. Black triangles indicate the interacting cell types derived from nonmalignant tissues. e) Diagrams of ligand‐receptor interactions in the four stages.

It is known that several pathways played a key role during tooth germ morphogenesis.^[^ ^12^ ^]^ So, we profiled the expression patterns of the key genes in these pathways in the ST. Co‐expression analyses showed that the morphogen modules from four mechanical pathways (MAPK, PI3K‐AKT, mTOR, and Hippo) and four classical pathways (TGF, WNT, SHH, and FGF) had crosstalk and interactions in 11 compartments (**Figure** **7**a). This finding indicated the involvement of mechanical signals in tooth morphogenesis, which corroborated the results in GO biological function enrichment and KEGG analysis. Our data also showed that the eight pathways’ modules in the dental germ layers had similar spatial and temporal distribution patterns. Specifically, they were enriched in the epithelium at the bud stage, in the mesenchyme at the cap stage, and at the epithelium‐mesenchyme interface at the bell stage and crown stage (Figure 7b). To validate the migration of dental embryonic epithelial cells under mechanical stress, we conducted an in vitro experiment. Ameloblast‐lineage cells were cultured above the trans well and subjected to 100 kpa pressure, resulting in a significant up‐regulation of their migration ability. This experiment confirmed that tooth germ epithelial cells can indeed accelerate their migration under stress. (Figure S13, Supporting Information) This transition pattern was consistent with the classical theory that the primary germinal center was located in the epithelium before switching to the mesenchyme.

To determine how the mechanical pathways were activated, we examined cell‐cell interactions at the epithelium‐mesenchyme interface by exploring the expression of ligand‐receptor pairs in dental germs (Figure 7c). We identified significant acting mode‐RTK (receptor tyrosine kinase) interactions, including VEGFD‐KDR, EGFR‐MIF2, MDK‐SOL1, PDGFRB‐PDGFR, PTN‐PTPRS, and LAMP1‐PAN3C, which continued to exist during tooth germ development.^[^ ^13^ ^]^ Additionally, the expression levels of the members of these RTK interactions were higher than those of the classical interactions (TGF‐TGFR, FGF‐FGFR, WNT5A‐PTPRK, and NOTCH2‐JAG) throughout odontogenesis. Cell surface RTKs could facilitate abundant cell‐to‐cell communication. In response to stimuli such as growth factors, these proteins phosphorylate their downstream cytoplasmic substrates. PTN,^[^ ^14^ ^]^ for example, is a cytokine and growth factor that promotes cell proliferation, adhesion, and migration by promoting receptor dimerization and tyrosine autophosphorylation.^[^ ^15^ ^]^ These RTK interactions have been linked to the activation of MAPK, PI3K‐AKT, mTOR, and Hippo mechanical pathways, corroborating the KEGG and Co‐expression analysis (Figure 7d). Furthermore, we summarized the interaction patterns and drew ligand‐receptor interaction diagrams of the four stages, indicating the key role of RTK interactions in regulating spatial compartment development (Figure 7e). We further performed the AFM to test stiffness of different compartments in ST. The stiffness of the epithelium‐mesenchyme interface especially cluster 8 was significantly higher than other clusters (Figure S14, Supporting Information). Taken together, we established the regulatory network directing tooth morphogenesis and displaced the coordinated epithelium‐mesenchyme interactome in time and space.

### 2.8 Charting the Cellular Basis of Congenital Tooth Disorders

**Figure 8:**
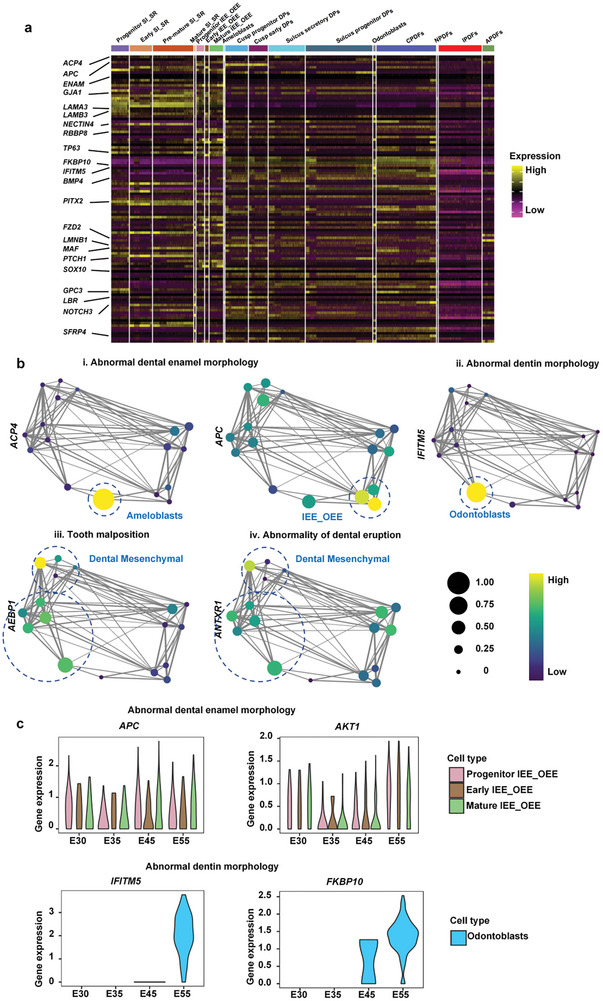
Applications of developmental transcriptional profiles in dental dysplasia. a) Heatmap representing the expression level of cell‐type specific dental dysplasia genes summarized from dental dysplasia HPO phenotype terms. b) Cell source analysis representing selected genes related to dental dysplasia. Graph abstraction overlay of cluster expression of disease genes associated with (i) abnormal dental enamel morphology (*APC4* and *APC*), (ii) abnormal dentin morphology (*IFITM5*), (iii) tooth malposition (*AEBP1*), and (iv) abnormal dental eruption (*ANTXR1*). c) Violin plots representing individual time‐course variations in dysplasia‐associated gene expression for abnormal dental enamel morphology and abnormal dentine morphology. *APC* and *AKT1* expression over time in IEE_OEE cells are shown above. *IFITM5* and *FKBP10* expression over time in odontoblasts are shown below.

Congenital developmental disorders can impose an extra burden on patients, both physically and psychologically. To explore the possible key transcription factors associated with congenital dental dysplasia, we performed a correlation study applying the list of dental developmental disorders from the human Phenotype Ontology (HPO) together with our data.^[^ ^16^ ^]^ We primarily connected the congenital disease phenotypes to possible cell‐type specific genes by integrating pathogenic genes with our single‐cell data (**Figure** **8**a). The results indicated that the *APC4* and *APC* genes, related to abnormal enamel morphology, were highly expressed in ameloblasts and matured IEE_OEE cells (Figure 8b(i)). The abnormal dentin morphology related *IFITM5* was specifically expressed in odontoblasts (Figure 8b(ii)). *ANTXR1* and *AEBP1*, associated with tooth malposition and dental eruption abnormality, were both highly expressed in the dental mesenchyme (Figure 8b(iii,iv)). The tooth number abnormality‐related genes *ACOX1* and *ACSL4* were prominently expressed in ameloblasts (Figure S15, Supporting Information). Furthermore, by temporal analysis, we further found that the expression levels of *APC* and *AKT1* were significantly increased at the E45 stage, suggesting E45 as a possible critical time point of abnormal enamel morphogenesis. The expression of *FKBP10* and *IFITM5* in odontoblasts was also increased at the E55 stage, implying E55 as a possible key time point of abnormal dentin morphology (Figure 8c). Thus, our data defined the major pathogenic cells and determinants linked to tooth dysplasia in time and space, providing new insights into disease occurrence and treatment.

## 3 Discussion

Understanding the lineage allocation, differentiation hierarchies, biological functions, and interaction networks of the different cell clusters that regulate tooth development is important to develop innovative regenerative strategies.^[^ ^17^ ^]^ To date, histology observation, genetic profiling, and niche ablating studies have significantly advanced this knowledge of tooth morphogenesis, but are confounded by the absence of high‐resolution spatial and temporal molecular information.^[^ ^18^ ^]^ Herein, we constructed a high‐resolution spatiotemporal framework for the molecular architecture of tooth morphogenesis by combining single‐cell sequencing with spatial transcriptomics analysis. Our data covered four major developmental stages: The bud stage, cap stage, bell stage, and crown stage (Figure 1). Thus, we provided insights into the coordinated emergence of different cell clusters, defined the regulatory networks manipulating their development, and charted the nature of their cross‐talk with neighboring cells.

It has been generally assumed that cell types continuously increase in the dental epithelium and mesenchyme during tooth morphogenesis.^[^ ^7^ ^]^ Our data showed that 88% of total lineage species with a determined cell‐fate have already appeared in the dental germ layers since the bud stage and are maintained until the bell stage (Figure S3a, Supporting Information). The increased heterogeneity during morphogenesis is not caused by increasing cell types, but relies on the varied cell distribution and cell proportion. The initially homogeneously distributed cell clusters are gradually grouped together by species to form distinct compartments. Meanwhile, the proportion of pluripotent cells gradually decreases during development, whereas that of differentiated cells gradually increases. Although Kaukua et al.^[^ ^2a^ ^]^ also found that the dental mesenchyme has multiple cell clusters at the bud stage, they still supported the traditional point of view that lineage divergence increases continuously during development. Our findings indicated that dental germs possess most of the functional cells required for morphogenesis from very early stage.

Another notable finding was the identification of a sprouting‐like patterning mode of cell clusters in the dental papilla during tooth morphogenesis. The dynamic structure of the dental mesenchyme was noticed by Michaela et al. through in vitro tissue culture combined with vital cell labeling and tissue grafting.^[^ ^19^ ^]^ Using single‐cell transcriptome profiling, Yang et al.^[^ ^20^ ^]^ reported that the two clusters in the dental papilla at the bell stage evolved to six clusters with significant spatial displacement at the crown stage. Meanwhile, Hong et al. found that *MSX1*+ *SOX9*+ cells might migrate upward after entering the bell stage to originate the dental papilla.^[^ ^21^ ^]^ However, the tissue organization of tooth morphogenesis was still confused in these studies. In this study, we provided a quantitative description of gene expression in whole developing dental germs, illustrating the lineage allocation in time and space. The coordinated lineage trajectory and spatiotemporal transcriptome analysis showed that the dental papilla at the bell stage was stratified to two layers, in which compartment 9 (cusp progenitor DPs and cusp early DPs) in the apical area was covered by the outer shell of compartment 8 (sulcus progenitor DPs and sulcus secretory DPs) in the coronal area. When entering the crown stage, compartment 9 evolved into a group of functionally graded cells, which broke through the cover of compartment 8 and occupied the cusp area of the papilla. Meanwhile, the primitive outer shell of compartment 8 migrated to the sulcus and sidewall of the papilla (Figure 2b). Such a patterning plan is significantly different from the previously established models, which suggested that the compartment localized at the papilla cusp area in early stages builds up the final papilla cusp.^[^ ^22^ ^]^ Our data revealed the sprouting‐like patterning mode of the dental papilla, which might advance our understanding of the life strategy driving the body plan.

Ameloblasts and odontoblasts are two highly differentiated cells with the capability to secrete enamel and dentin, respectively, for tooth structure mineralization. Developmental biology research in the past 60 years has taught us that these two cells appear at the bell stage. According to Hermans et al,^[^ ^23a^ ^]^ the ameloblasts and odontoblasts that emerged at the bell stage were premature clusters. Our findings also indicated that matured ameloblasts and odontoblasts appeared at the crown stage.^[^ ^23^ ^]^ We found that both of these mature cells first appeared at the crown stage when the framework of tooth morphology is almost established (Figures 4 and 5). The developmental trajectory indicated that ameloblasts with high expression of Enamelin are derived from matured IEE_OEE cells, while odontoblasts with high expression of *DMP1* originated from cusp progenitor cells. The spatial distribution diagram of single cells showed that the traditionally considered ameloblasts are actually matured IEE_OEE cells, which have high expression of *Ablim1* to facilitate cytoskeleton organization (Figure 4). These cells were localized at the epithelium‐mesenchyme interface to drive epithelium migration around the dental papilla for morphogenesis. Similarly, in contrast to the traditionally held view, we revealed that odontoblasts are actually cusp progenitor DPs, which have high expression of matrix mineralization *VCAN*. The sulcus secretory DPs were localized in the cusp area of the dental papilla adjacent to enamel knots at all stages, supporting a complicated inductive interaction between sulcus secretory DPs and enamel knots.^[^ ^24^ ^]^ Then, sulcus secretory DPs finally migrated to the side wall and sulcus, while the real odontoblasts sprout out to the cusp area of the papilla at the crown stage (Figure 5F). The appearance of odontoblasts at the crown stage was observed recently by Yang et al.^[^ ^20^ ^]^ Nevertheless, they reported that odontoblasts originated from apical papilla cells, in contrast to our results. This difference could be accounted for by the fact that their sample collection time at the crown stage was much later than ours because the tooth enamel formed in their study was much thicker than that in the present study. At that time, the spatial area of the apical papilla cells was actually occupied by combinations of cusp progenitor DPs and sulcus progenitor DPs. The illustration of the spatiotemporal lineage trajectory of ameloblasts and odontoblasts redefined the time point when the mineralization of the tooth structure is first launched.

As the signal center of tooth development, enamel knots were revealed at the local thickness of the inner enamel epithelium (IEE) with high expression of *SHH*, *FGF4*, *BMP4*, and *WNT* to orchestrate morphogenesis.^[^ ^25^ ^]^ However, the lineage history and segregation of enamel knots in development and their location dimension remained unknown. Using a genome‐wide molecular annotation of dental germ tissue architecture, we hypothesized that the enamel knot was an intermediate stage of the IEE‐OEE cluster. The enamel knots (early OEE‐OEE cells), their precursors (progenitor OEE‐OEE cells), and their offspring (mature OEE‐OEE cells) have already emerged in the dental epithelium during the bud and cap stages.^[^ ^16^ ^]^ The precursors of enamel knots were distributed in the whole epithelium and had a high density in areas proximal to the epithelium‐mesenchyme interface at all stages (Figure 4g). With high expression of mitosis‐related *CENPF*, these precursors could serve as the key source for epithelial proliferation. The early IEE_OEE cells had characteristics consistent with classical description of enamel knots, which were located at the cusp‐tip area of the epithelium‐mesenchyme interface and showed high expression of morphogenesis‐related signaling molecules, including *WNT10b*, *FGF9*, and *SHH*. The offspring cluster was localized at the epithelium‐mesenchyme interface lateral to the early OEE‐OEE cells. These cells had a highly organized cytoskeleton to facilitate epithelial migration for morphogenesis and would give rise to ameloblasts for mineralization. The defined continuum differentiation hierarchies of enamel knots in our study would help to trace the dynamic variation of the signal center in tooth development.

Additionally, we identified several previously unrecognized but important cells in dental germs. In the epithelium, *KLF4*+ progenitors cells distributed in the coronal tip area of the epithelium were observed from the bud stage to the bell stage (Figure 4e). With high expression of growth factor‐related *IGFBP5*, proliferation‐related *PDGFRB*, and stem cell marker *KLF4*, these cells might provide essential pluripotency for epithelium differentiation. In the dental follicle, we identified an immune progenitor cluster (immune progenitor denticle follicle cells (IPDFs)) with high expression of immunomodulation‐related *C1QB*, *ATF5IF1*, and *NFKBIA*, and vascular progenitor cluster (angioblast progenitor denticle follicle cells (APDFs)) with high expression of vascularization‐related *EFGL7*, *PECAM1*, *ESAM* (Figure 6). These cells were almost evenly distributed over the dental follicle at all stages, indicating their supportive role in periodontal tissue generation. In the apical area of the dental papilla and follicle, Cusp progenitor DPs and neural progenitor denticle follicle cells (NPDFs) with high expression of neurogenesis‐related genes were identified, which would differentiate into nerve tissues in the pulp and periodontal tissue. Our findings might provide potential new seeding cells with delicate functions for tooth regeneration strategies.

Biological research in the past decades has established that tooth development is dominated by the classical odontogenesis pathways, including the TGF‐β, WNT, SHH, and FGF pathways.^[^ ^26^ ^]^ By investigating scRNA‐seq profiling of epithelial and mesenchymal cells functional states and the expression of various ligand‐receptor pairs among single cells, we established the regulatory signaling network directing tooth morphogenesis. Our results indicated that mechanotransduction signals are involved in regulating tooth morphogenesis beyond the classical odontogenesis pathways. The graphical visualization of the interactome of the morphogen molecule STRING identified 11 cell‐type‐specific and spatially co‐localized morphogen modules, suggesting that the classical odontogenesis pathways and mechanotransduction pathways of MAPK, Hippo, PI3K‐AKT, and mTOR^[^ ^27^ ^]^ constituted a complicated regulatory network that orchestrates tooth morphogenesis (Figure 7a). The enrichment analysis of signaling target genes also indicated that these eight pathways had similar spatiotemporal activity patterns in dental germ layers, which were exclusively enriched in the epithelium at the bud stage, in the mesenchyme at the cap stage, and at the epithelium‐mesenchyme interface at the bell and crown stages. Such a temporally varied spatial distribution was inconsistent with the established signal transmission theory,^[^ ^16^ ^]^ in which the signal center was initially localized in the dental epithelium and later transferred to the mesenchyme during development. In addition, we found that the activity of the eight pathways was significantly enriched at the epithelium‐mesenchyme interface at the morphogenesis milestone of the bell and crown stages, suggesting their importance in manipulating tooth shape. The activation of mechanotransduction events in dental germs could be ascribed to the interactome of a series of upstream RTKs between cell clusters that were constantly activated during tooth development. Taken together, these findings implied the indispensable role of biomechanical events in tooth morphogenesis and provide potential regulatory targets.

Furthermore, based on the high‐resolution molecular and cellular resources across tooth development.^[^ ^28^ ^]^ we defined the major pathogenic cells and determinants in time and space linked to tooth dysplasia disease (Figure 8). Abnormal enamel morphology and abnormal dentin morphology are the two major types of tooth developmental dysplasia. Previous studies have targeted several candidate genes of these diseases; however, their pathogenesis has remained elusive given the undetermined mutation time and cell location.^[^ ^29^ ^]^ Here, we correlated our data with a curated list of tooth dysplasia diseases from the Human Phenotype Ontology (HPO), annotated with hereditary phenotypes, and successfully tracked genes linked to these genetic defects to highly specific time points and cell types. We highlighted that *ACP4* gene mutation in ameloblasts and *ACP* mutation in matured IEE_OEE cells at the crown stage are linked with abnormal enamel morphology. Similarly, pathogenic mutants/variants of the bone‐restricted interferon‐induced transmembrane gene *IFITM5* in odontoblasts at the bell stage could lead to abnormal dentin morphology. Thus, our findings regarding tooth morphology dysplasia diseases will pave the way for the development of specifically targeted genetic therapy.

Taken together, by combining a spatial transcriptome with single‐cell RNA‐seq, we described the high‐resolution genome‐wide molecular patterning of lineage allocation and cellular structure in tooth morphogenesis. We found that 88% of all lineage species had already made their appearance in the initial tooth bud stage, indicating that the dental germs possess most of the functional cells required for morphogenesis from very early stage. Our data revealed a sprouting‐like patterning mode of the dental papilla that the inner compartment could break through the outer‐shell compartment to occupy the papilla cusp, which is significantly different from the previously established theory that the compartment localized at the papilla cusp area at early stages build up the final papilla cusp. This finding would advance our understanding of the life strategy driving the body plan. We found that ameloblasts and odontoblasts made their appearance at the crown stage rather than the previously considered bell stage, which redefined the time point when the mineralization of the tooth structure was first launched. We also illustrated the continuum differentiation hierarchies of enamel knots in time and space, which could help to trace the dynamic variation of the signal center in tooth development. Furthermore, we established the regulatory signaling network directing epithelium‐mesenchyme interactions. Mechanotransduction signals were revealed to be significantly involved in tooth morphogenesis beyond the well‐established classical odontogenesis signals. This result raised the idea that tooth morphogenesis was orchestrated by mechanical niches combined with biochemical signaling. Finally, based on the genetic structure of tooth morphogenesis established in our study, we defined the major pathogenic cells and determinants in time and space that are linked to tooth dysplasia. Our work detailed comprehension of the sub‐atomic engineering of tooth morphogenesis offers a mechanistic starting point for possible hereditary control.

## 4 Experimental Section

### Pig Strains and Animal Care

All animals used in the experiments were supplied by the Beijing Shichuangshiji miniature pig breeding farm. The study was approved by the Beijing Shichuang Century Small Pig Breeding Base Ethics Committee and carried out according to the principles of laboratory animal ethics and welfare, project number: SC2020‐08‐008).

### Sample Collection and Cell Preparation for scRNA‐Seq

The tooth germs (E30, *n* = 2; E35, *n* = 4; E45, *n* = 4, E55, *n* = 2) were physically separated from mandibular using microsurgical instruments, Tissues surrounded the tooth dental were carefully removed, and collected into a clean culture plate. After the tooth dental were washed twice with 1xPBS, the tissues were minced into ≈1 mm^3^ fragments with sharp scissors and digested with collagenaseII(Solarbio, C8150, 2 mg mL^−1^,) and DNase I(10 ug mL^−1^) at 37 °C for 1 h, then, the single cell suspensions were collected through a 40 um strainer, and centrifuged at 500 g for 5 min, the supernatant was completely aspirated and cells were resuspended, finally, the cell number and viability were assessed by an automatic cell counter.

### Construction of Single‐Cell RNA Library and Sequencing

Using a single cell 3 ′Library and Gel Bead Kit V3.1 (10x Genomics, 1000121) and Chromium Single Cell G Chip Kit (10x Genomics, 1000120), the cell suspension (300‐600 living cells per microliter determined by Count Star) was loaded onto the Chromium single cell controller (10x Genomics) to generate single‐cell gel beads in the emulsion(GEMs) according to the manufacturer's protocol. In short, single cells were suspended in PBS containing 0.04% BSA. ≈15 000 cells were added to each channel, and ≈10 000 cells were recovered. Captured cells were lysed and the released RNA was barcoded through reverse transcription in individual GEMs. Reverse transcription was performed on a S1000TM Touch Thermal Cycler (Bio Rad) at 53 °C for 45 min, followed by 85 °C for 5 min, and held at 4 °C. The cDNA was generated and amplified, finally quality was assessed using an Agilent 4200 (performed by CapitalBio Technology, Beijing).

According to the manufacture's introduction, Single‐cell RNA‐seq libraries were constructed using Single Cell 3′ Library and Gel Bead Kit V3.1. The libraries were finally sequenced using an Illumina Novaseq6000 sequencer with a sequencing depth of at least 50 000 reads per cell with pair‐end 150 bp (performed by CapitalBio Technology, Beijing).

### Single‐Cell RNA Sequencing Data Preprocessing

Raw gene expression matrics were generated for each sample by Cell Ranger(4.0.0) with default. Each sample was aligned to (Sscrofa11.1). Then, the matrics were converted into a Seurat object by the R package Seurat v4.0.0. Cells with gene numbers <200, mitochondrial gene ratio >25% or gene number ranked in the top 1% or were regarded as low‐quality cells and filtered out. The filtered data were normalized using “NormalizedData” function. Dimensionality reduction was performed using PCA, and visualization was realized by UMAP. Then, the Harmony package was used to integrate data from different samples. A publicly available online resource, spatio‐temporal analysis resource of dental germ development (STAR‐FINDer), is compiled to facilitate further work (GSA ID CRA015396).

### Cell Type Annotation and Marker Cluster Identification

Cell type was annotated by matching each cluster‐specific gene with known signature genes of cell populations in previous literatures.

### Differential Gene Expression and Function Enrichment

Differential gene expression testing was performed using FindMarkers function in Seurat with parameter “test.use = wilcox” and the Bonferroni multiple testing adjustment was used to estimate the false discovery rate(FDR). DEGs were filtered using log2FC > 1 and adjusted *p*‐value < 0.05. GO and KEGG enrichment analysis of significant DEGs were then performed. The results were visualized using R package.

### Single‐Cell Trajectories Analysis

Single‐cell trajectories were constructed using Monocle2 (R package) to determine the dramatic developmental trajectory and translational relationships. Significantly genes that expressed in more than ten cells with average expression value > 0.1 or adjusted *p*‐value (Q_val) < 0.01 were identified by the differential GeneTest function in Monocle2.

### Cell‐Cell Communication Analysis

Cell Phone DB2 was used to evaluate the cell‐cell communication in E30, E35, E45, 45 datasets. Interaction pairs including chemokine/cytokine and their receptors were identified.

### Sample Preparation for Spatial Transcriptome

The mandible tissues from miniature pigs (E30, *n* = 2; E35, *n* = 4; E45, *n* = 4; E55, *n* = 2) were snap‐frozen with precooled isopentane and then embedded with O.C.T. compound. Cryosections were cut with 10 µm thickness and mounted onto the GEX arrays. Sections were placed on Thermocycler Adaptor with the active surface facing up and incubated 1 min at 37 °C, and then were fixed for 30 min with methyl alcohol in −20 °C followed by staining with H&E (Eosin, Dako CS701, Hematoxylin Dako S3309, bluing buffer CS702).54 The brightfield images were taken via a Leica DMI8 whole‐slide scanner at ×10 resolution.

### Permeabilization and Reverse Transcription

Visum spatial gene expression was processed using the Visum spatial gene expression slide and Reagent Kit (10x Genomics, PN‐1000184). For each well, Slide Cassette was used to create leakproof wells for adding reagents. 70 µL Permeabilization enzyme were added and incubated at 37 °C for 30 min. Each well was washed with 100 µL SSC, and 75 µL reverse transcription Master Mix was added for cDNA Synthesis.

### cDNA Library Preparation for Sequencing

At the end of first‐strand synthesis, remove RT Master Mix from the wells. Add 75 µL 0.08 m KOH and incubate 5 min at room temperature, then remove the KOH from wells and washed with 100 uL EB buffer. Add 75 µL Second Strand Mix to each well for second‐strand synthesis. cDNA amplication was performed on a S1000TM Touch Thermal Cycler (Bio Rad) According to the manufacture's introduction.

Visum spatial libraries were constructed using Visum spatial Library construction kit (10x Genomics, PN‐1000184). The libraries were finally sequenced using an Illumina Novaseq6000 sequencer with a sequencing depth of at least 100 000 reads per spot with pair‐end 150 bp (PE150) reading strategy (performed by CapitalBio Technology, Beijing)

### Spatial Transcriptome Sequencing Data Processing

Alignment, filtering, barcode counting, and UMI counting were performed with Spaceranger (1.1.0) count module to generate feature‐barcode matrix and determine. Then, the data analysis was conducted using Seurat v4.0.0 (R package). Dimensionality reduction was performed using PCA, and visualization was realized by UMAP. The resolution of clustering was 0.6.

### Deconvolute Spatial Transcriptomics Spots with Single‐Cell Transcriptomes: Spotlight

After cell types annotation, scRNA‐seq as reference data set into our spatial profile via SPOTlight v0.1.7 to determine the proportions of cell types in each spot was mapped. SPOTlight learns topic signatures from a reference scRNA‐seq dataset and uses this to find the optimal weighted combinations of cell types to deconvolute the data from Visium spots into underlying cell type. 10X Visium data was deconvoluted by the spotlight_deconvolution function based on non‐negative matrix factorization (NMF). Eventually, the proportion of cell types in each spot was visualized by the spatial_scatterpie function. Default parameter settings were used for deconvolution analysis.

### Immunohistochemistry and Antibodies

Tooth germs were separated from eight miniature mice for immunofluorescence marker validation. The experiments were approved by the Biomedical Ethics Committee of Peking University and carried out according to the principles of laboratory animal ethics and welfare, project number: La2020452).

Samples were fixed with 4% paraformaldehyde (PFA), cryopreserved with sucrose, embedded in optimal cutting temperature (OCT) and transversely sectioned at 14 µm. Samples were brought to room temperature (RT), dried, and subjected to antigen retrieval using 1× Dako Target Retrieval solution (Dako, S1699). Slides were washed three times with PBS containing 0.1% Tween‐20 (Sigma, St. Louis, MO, USA; P9416) (PBST) for 10 min and incubated with primary antibody solution in PBST in a humidified chamber at RT overnight. Next day, the slides were washed three times with PBST for 10 min and incubated with secondary antibody solution and DAPI (1:10000; Thermo Fisher Scientific, Waltham, MA, USA; D1306) in PBST for 90 min, followed by three washes with PBST for 10 min at room temperature. Slides were mounted with Mowiol (Sigma, 81381). The following primary antibodies were used at the indicated dilutions: rabbit anti‐KLF4 (1:200, Abcam, Cambridge, MA, USA; ab215036), anti‐DSG1 (1:200, Abcam, ab124798), anti‐COL III (1:200, Abcam, ab184993), anti‐CENPF (1:250, Abcam, ab223847), anti‐Shh (1:200, Abcam, ab240438), rabbit anti‐CST3 (1:200, Abcam, ab109508), Alexa Fluor 488 (1:500, Abcam, ab150077 and ab 150129).

### Tooth Germ Stiffness

The tooth germ tissue section was examined using an atomic force microscope (Bruker). The PF θ nm‐lc‐a‐cal, LC4 probe was utilized with the following parameters: sum = 4.1 V (25.4 °C), calibrated with no touch, k = 0.104 n m^−1^, Def.sens. = 23.67 nm V^−1^. The probe parameter settings were as follows: Tip half angle = 18°, Poisson's ratio = 0.5, scan size = 500 nm (if there was no scanning morphology, it can be set larger to ensure that the X rotation in the ramp can reach 12°), peak force frequency = 1 kHz, peak force amplitude = 300 nm, engage setting amplitude = 300 nm. Ramp parameters included a ramp size of 1.5 µm; ramp rate of 1 Hz; X rotate of 12°; Z closed loop open; relative contact mode; and contact range of 6 nm.

## Conflict of Interest

The authors declare no conflict of interest.

## Supporting information



Supporting Information

## Data Availability

The data that support the findings of this study are available from the corresponding author upon reasonable request.
