# Supplementary material for: Spatiotemporal Molecular Architecture of Lineage Allocation and Cellular Organization in Tooth Morphogenesis
Source: Adv Sci (Weinh). 2024 Nov 13;11(47):2403627. doi: 10.1002/advs.202403627 (PMC11653630; doi:10.1002/advs.202403627)
Supplement: Supplementary file 1 — Supporting Information [file ADVS-11-2403627-s001.docx]

Supplementary Materials for

**Spatial and single-cell landscape of tooth morphogenesis**

Shengjie Jiang *et al.*

*Corresponding author. Email: [laib@bjmu.edu.cn;](mailto:laib@bjmu.edu.cn;) kqdengxuliang@bjmu.edu.cn;

kqweiyan@bjmu.edu.cn;

**This PDF file includes:**

Figs. S1 to S14


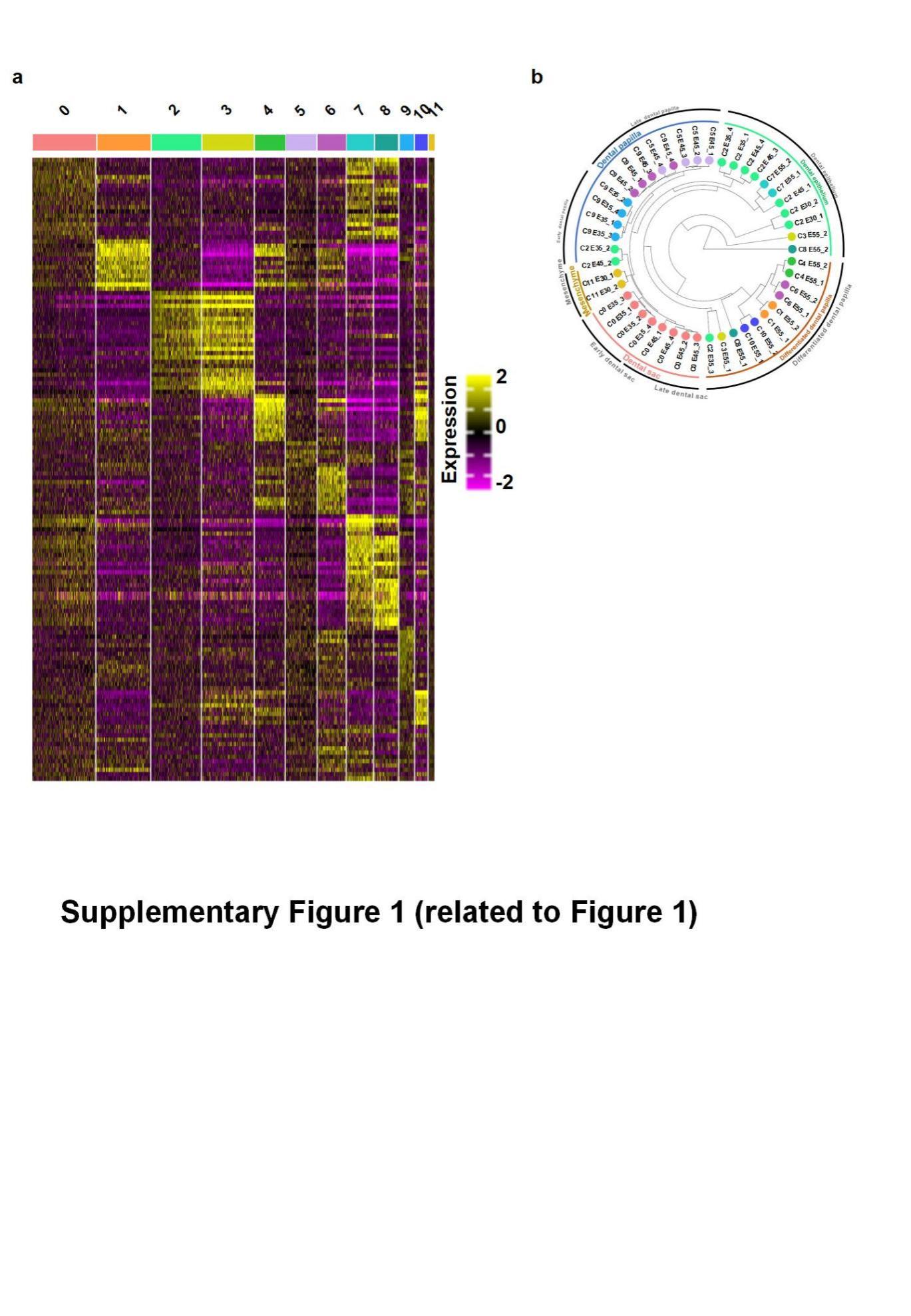


Figure S1. ScRNA-seq distinguishes major teeth germ compartments, related to Figure 1. Heat map analysis showing differential peaks of gene expression among each cluster.

.


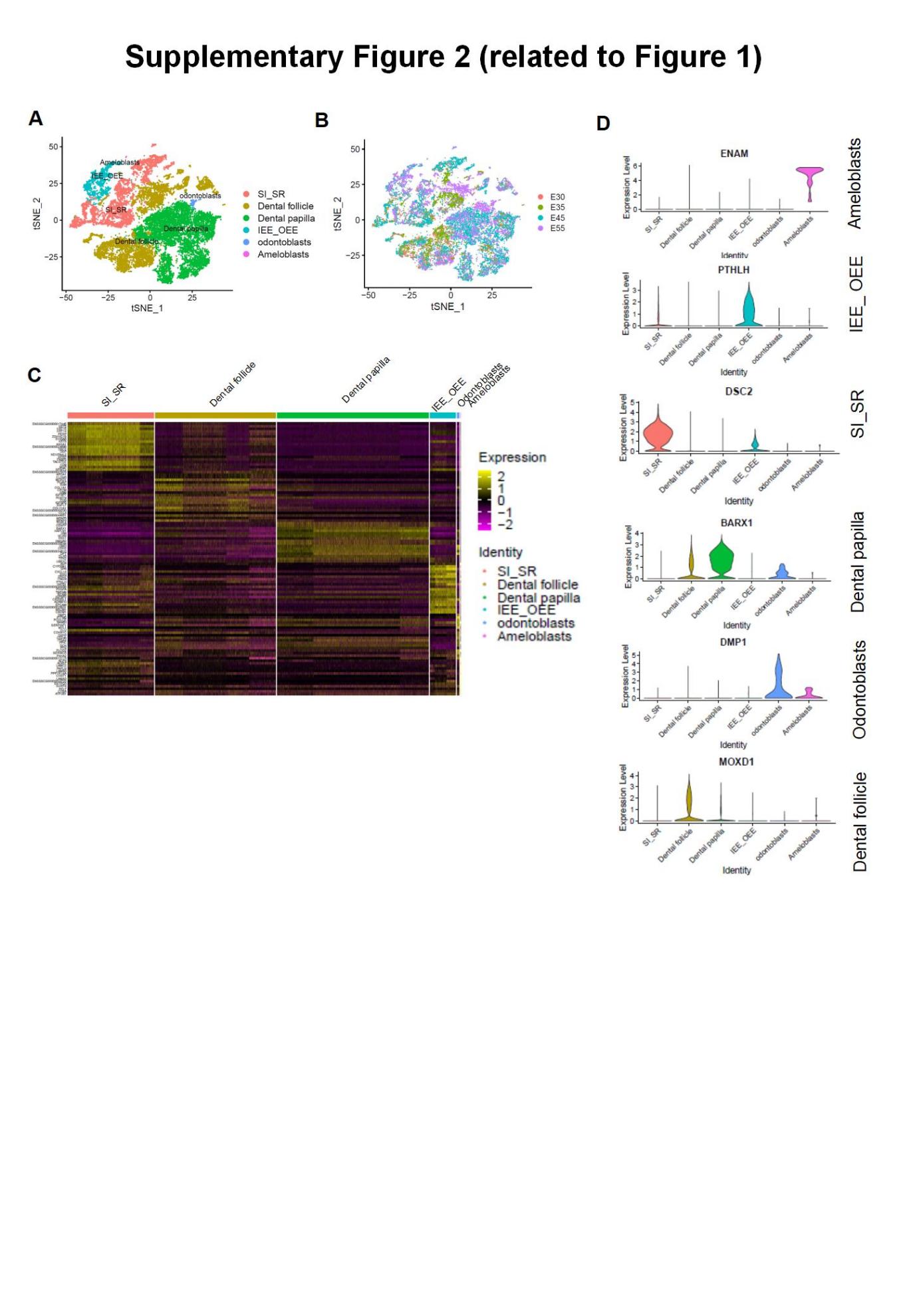


Figure S2. Six major clusters were identified for tooth germ, related to Figure 1. a-b A T-SNE plot showing six clusters within the dental germ (a) and the distribution based on the developmental time course (b). c Heat map analysis showing that each cluster possessed specific highly expressed genes. d Violin plot showing the expression level of specific marker genes closely related to epithelium and mesenchyme function.


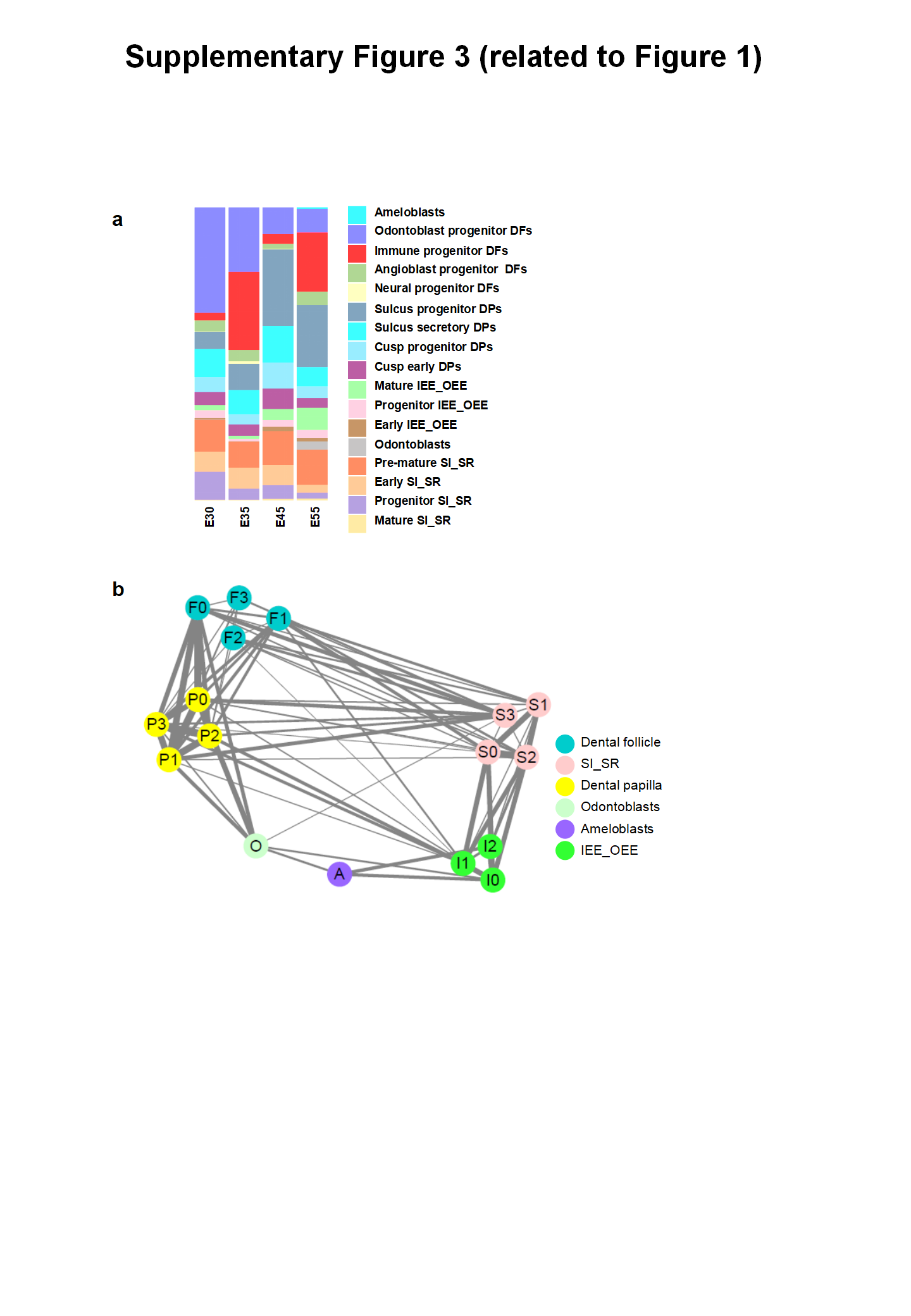


Figure S3. The proportional distribution of 17 clusters, related to Figure 1. a Bar plot showing the proportional distribution of the identified single-cell clusters in four developmental stages, indicating that 88% of the total lineage species with determined cell-fate have already appeared since the bud stage. b Partition-based graph abstraction of 17 clusters identified in the scRNA-seq data, colored by six major clusters.


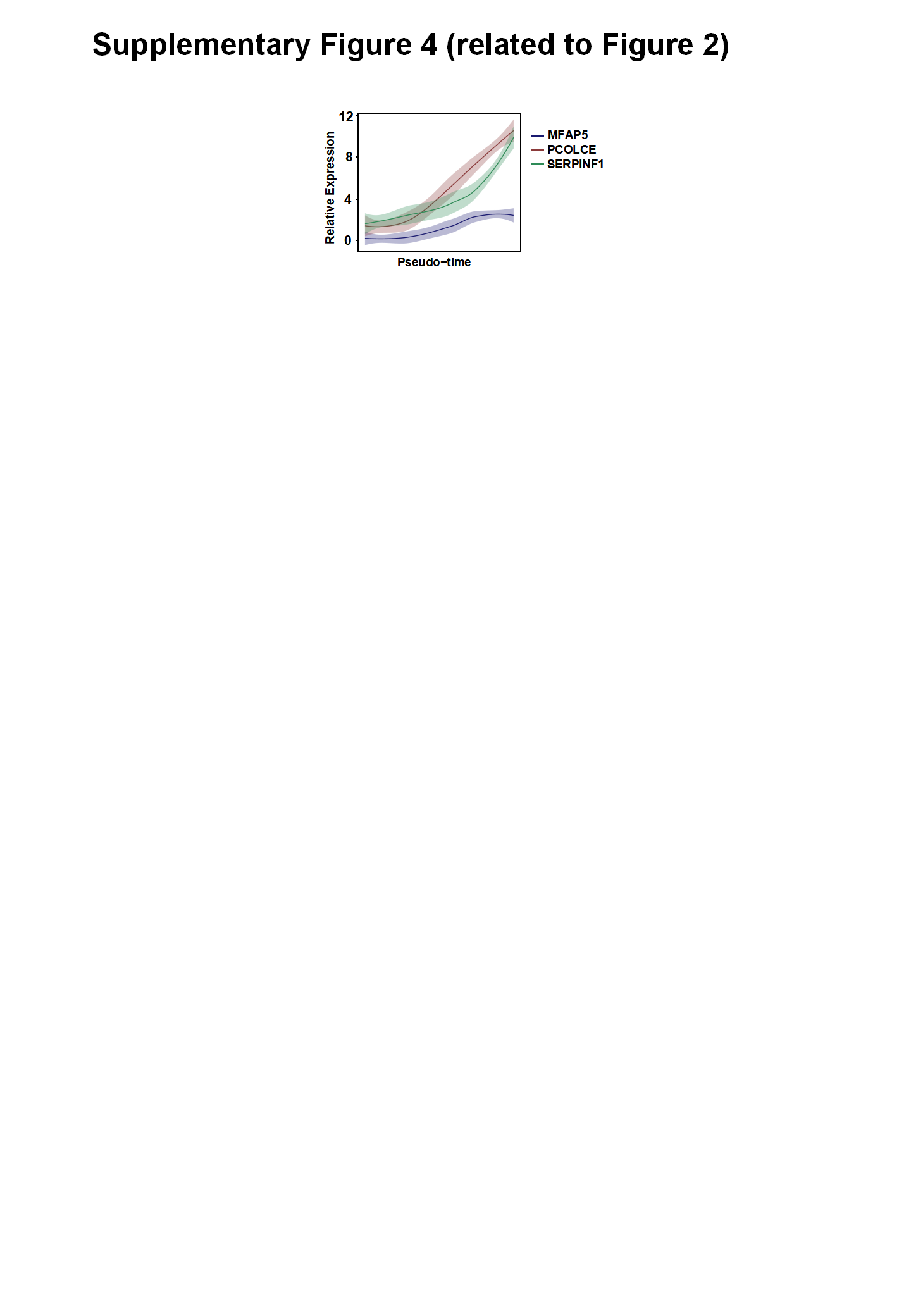


Figure S4. Epithelial pseudotime trajectory related to Figure 2. Dynamics of the genes involved in dental epithelial development along the pseudotime trajectory in Fig. 2D.


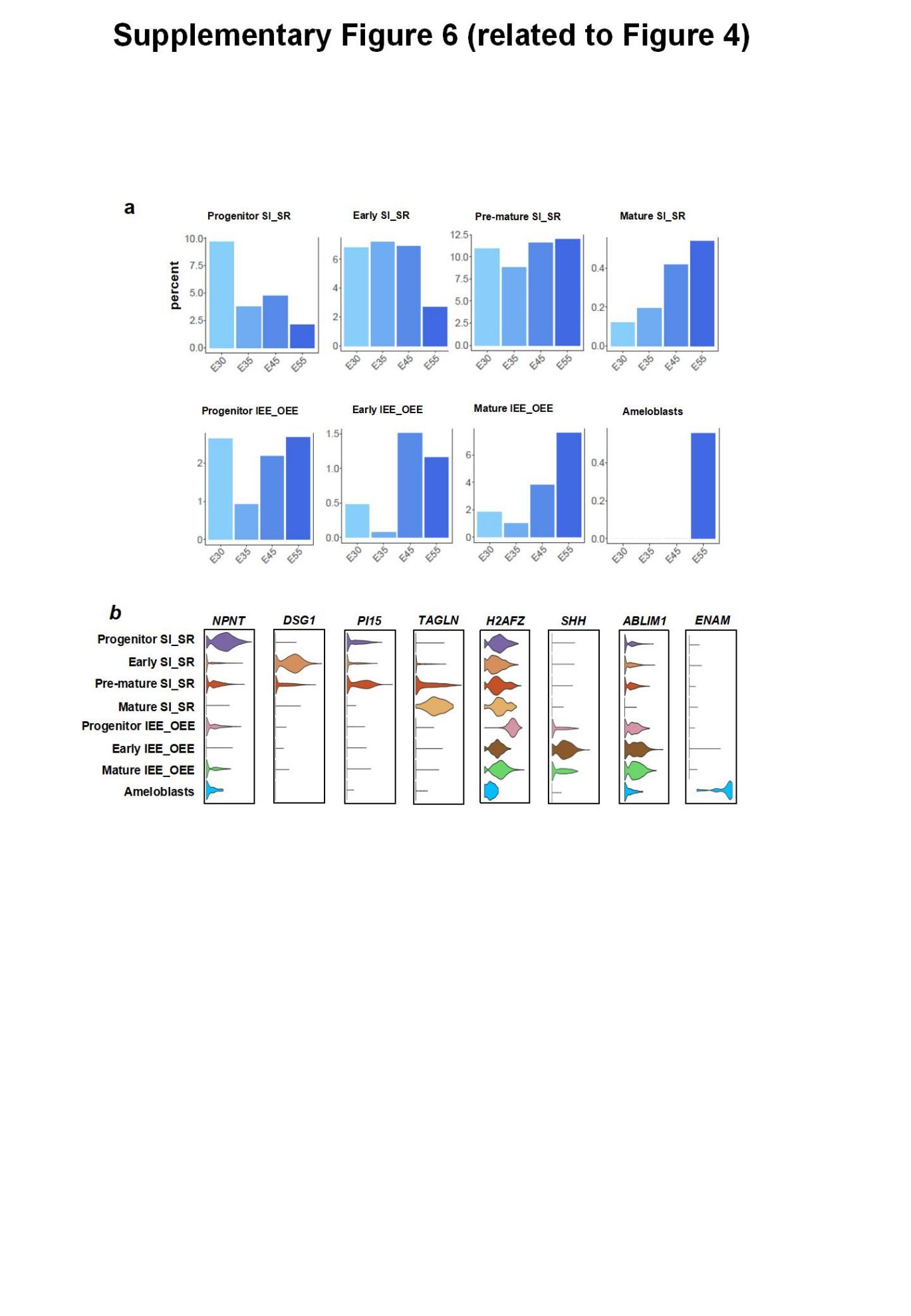


Figure S5. Dental germ epithelial development, related to Figure 4. a Bar plots showing the time distribution characteristics of dental papilla populations, indicating the occurrence sequence of progenitor clusters and differentiated clusters. b Violin plots representing the expression levels of specific marker genes closely related to epithelium functions and developmental mechanics.


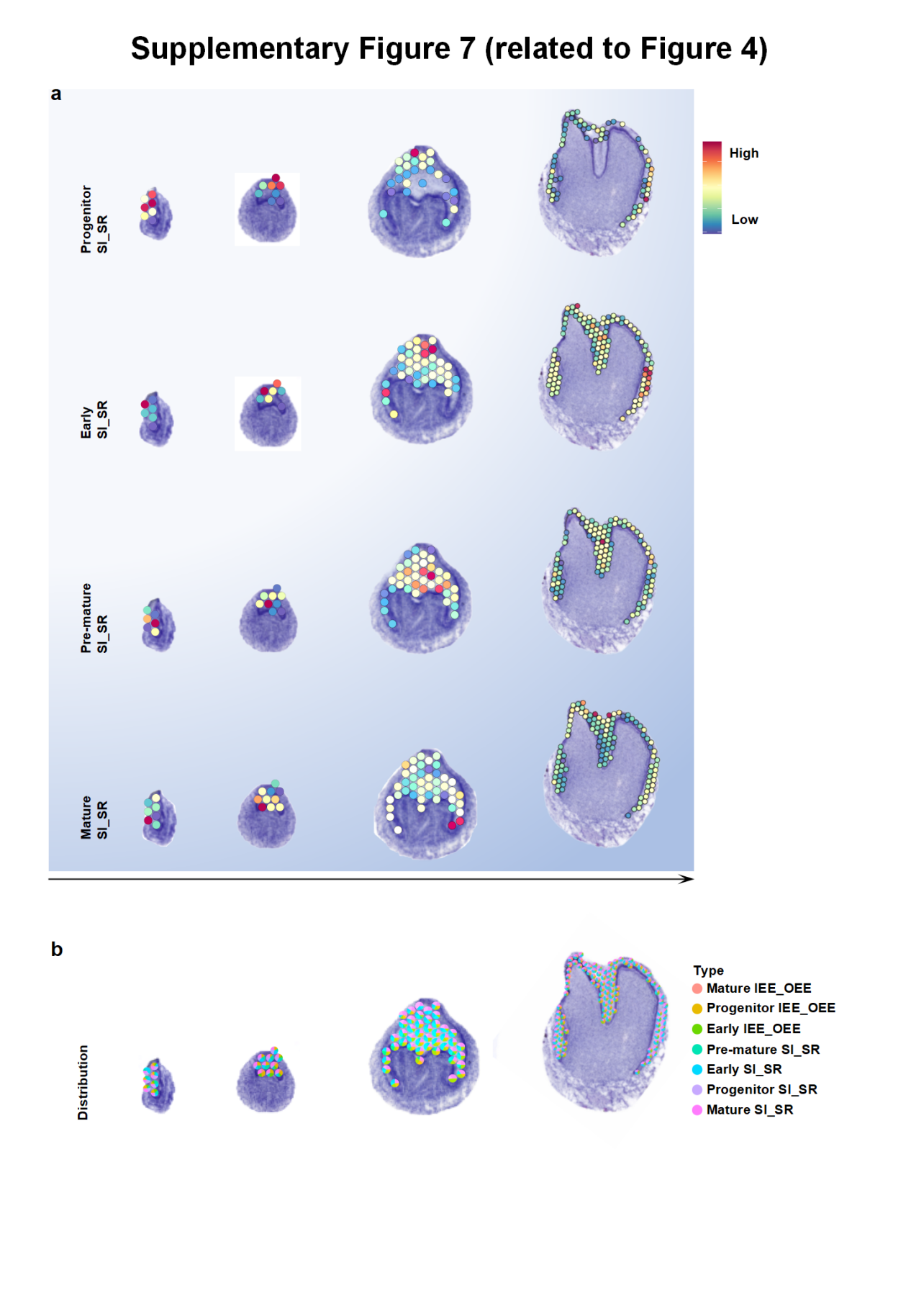


Figure S6. Dental germ epithelial spatial distribution, related to Figure 4. a-b Conjoint analysis of single cell and spatial transcriptome sequencing showing the evolution(a) and spatial distributions(b) of dental epithelial SI_SR clusters at the bud stage, cap stage, bell stage and the differentiation stage.


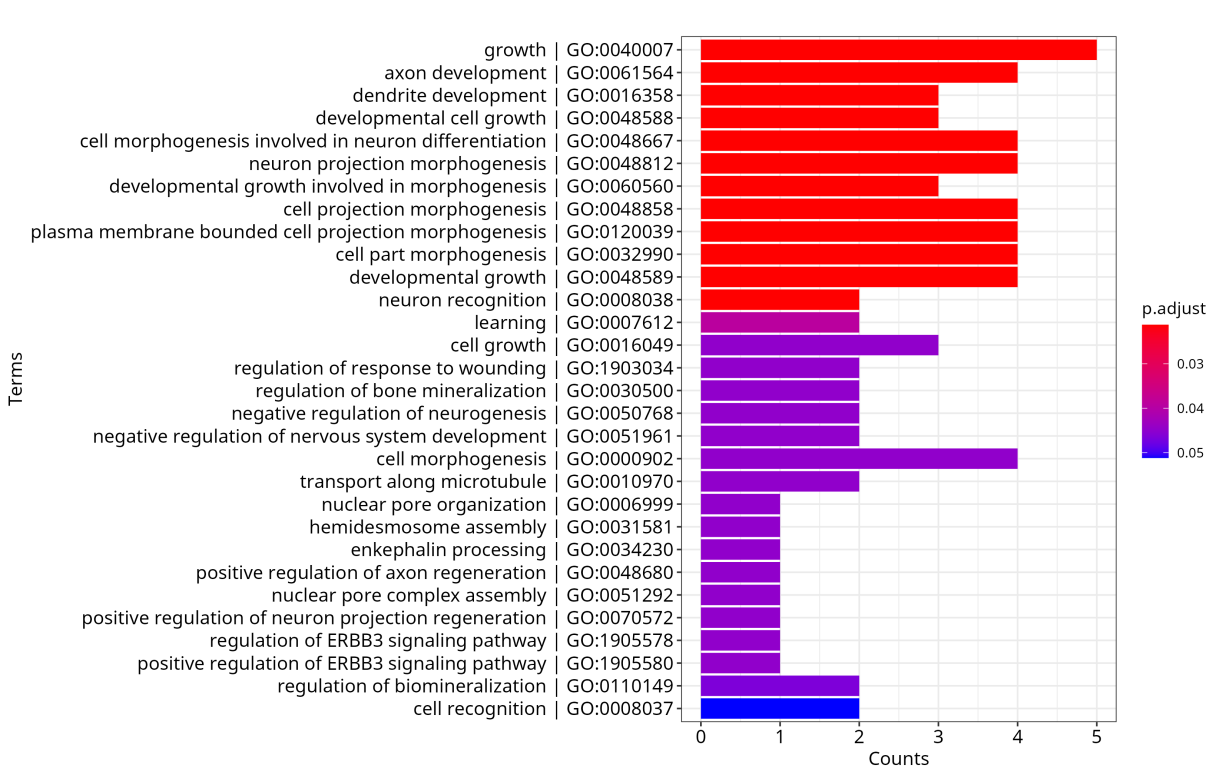


Figure S7. GO analysis on KLF4+ cluster, related to Figure 4.


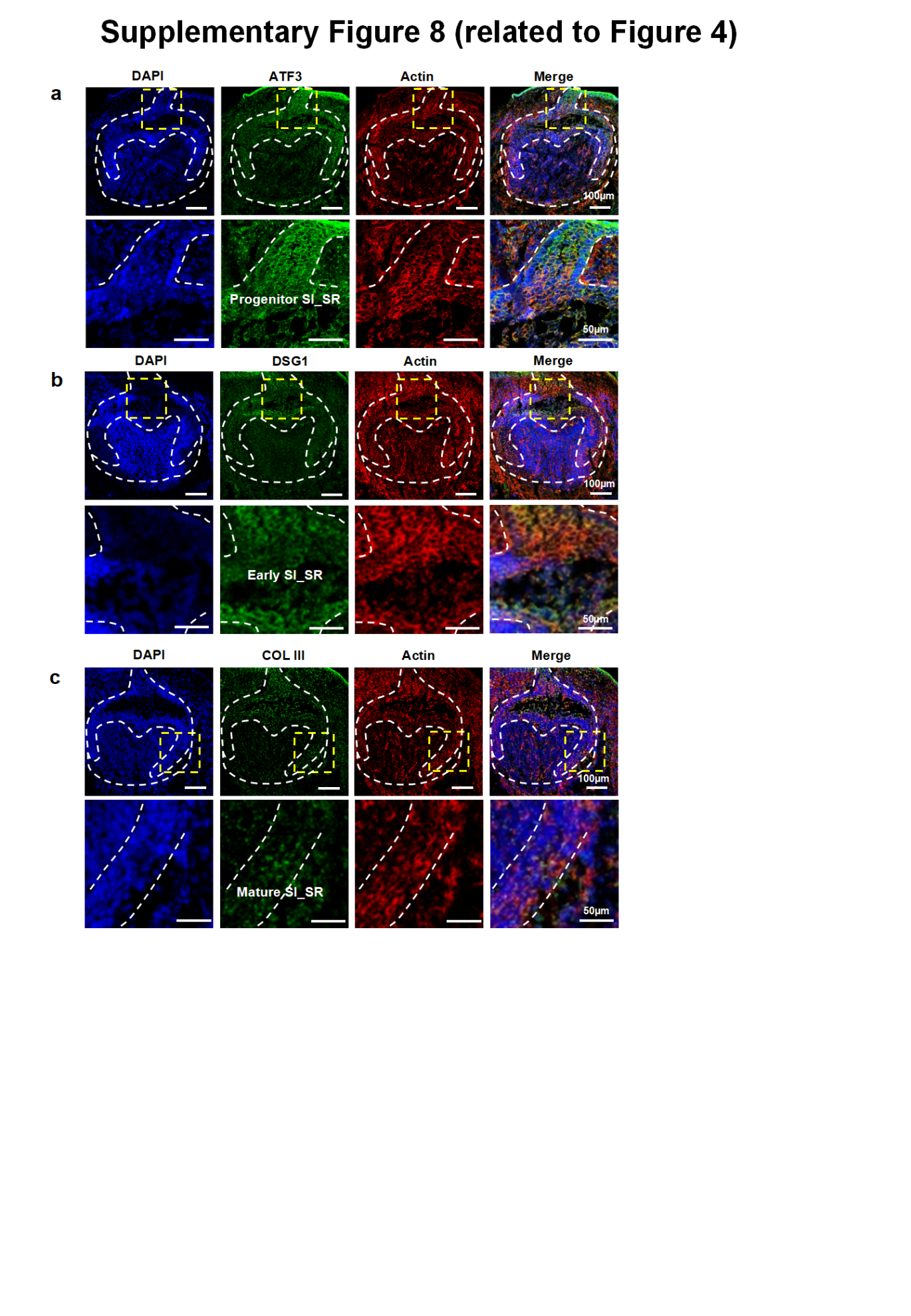


Figure S8. Spatiotemporal analysis of dental epithelial development by immunofluorescence staining, related to Figure 4

**a**-**c** Immunofluorescence staining visualizing the expression of ATF3 (**a**), DSG1 (**b**), and COL Ⅲ (**c**) in bell stage sections. Scale bars, 100 µm in the wide-fields; 50 µm in the insets.
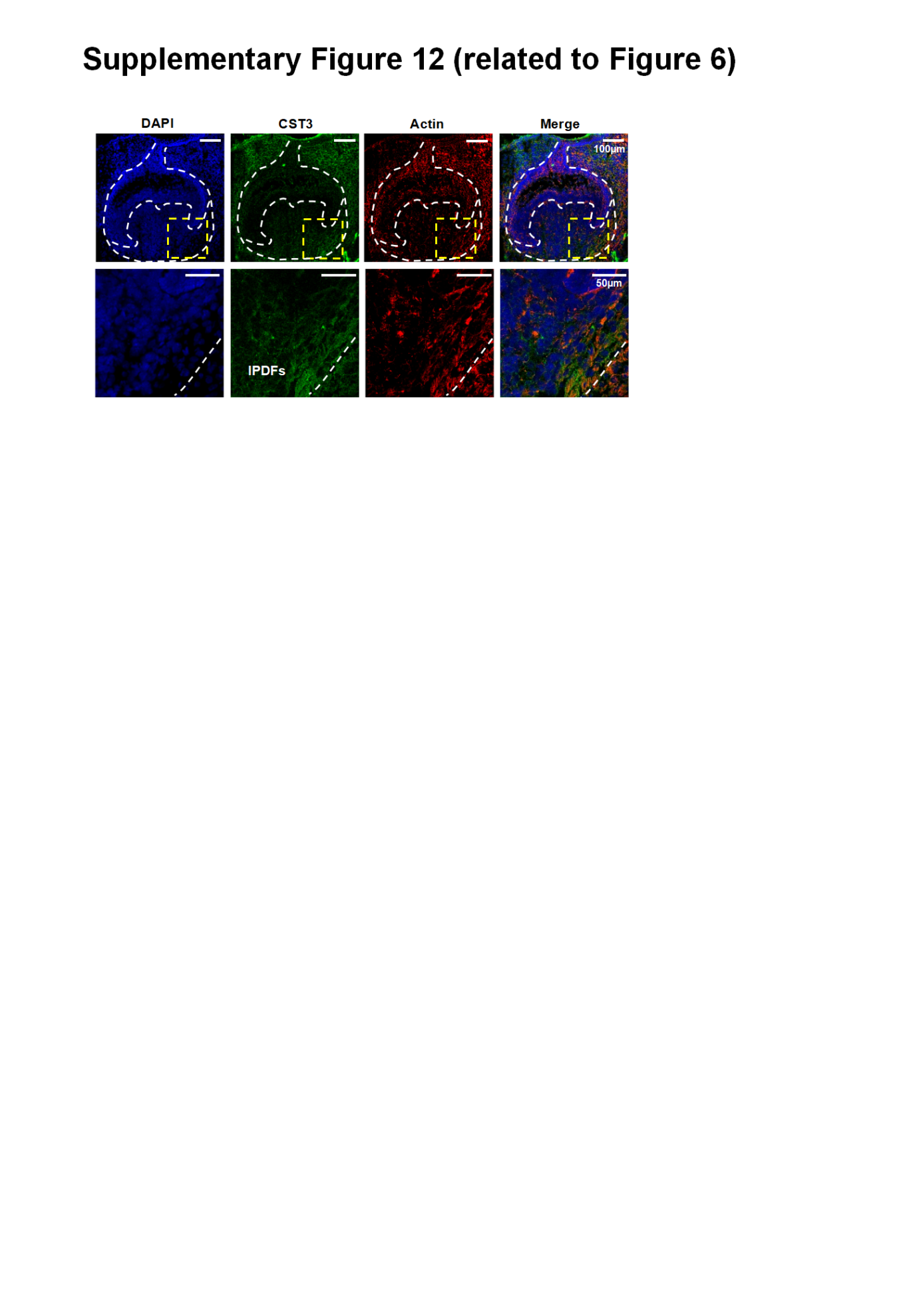


Figure S9. Spatiotemporal analysis of enamel knots by immunofluorescence staining, related to Figure 4.

**a** Immunofluorescence staining visualizing the main expression of CENPF in progenitor IEE_OEE cells. **b** Immunofluorescence staining visualizing the main expression of enamel knot marker SHH in early IEE_OEE cells. Scale bars, 100 µm in the wide-fields; 50 µm in the insets.
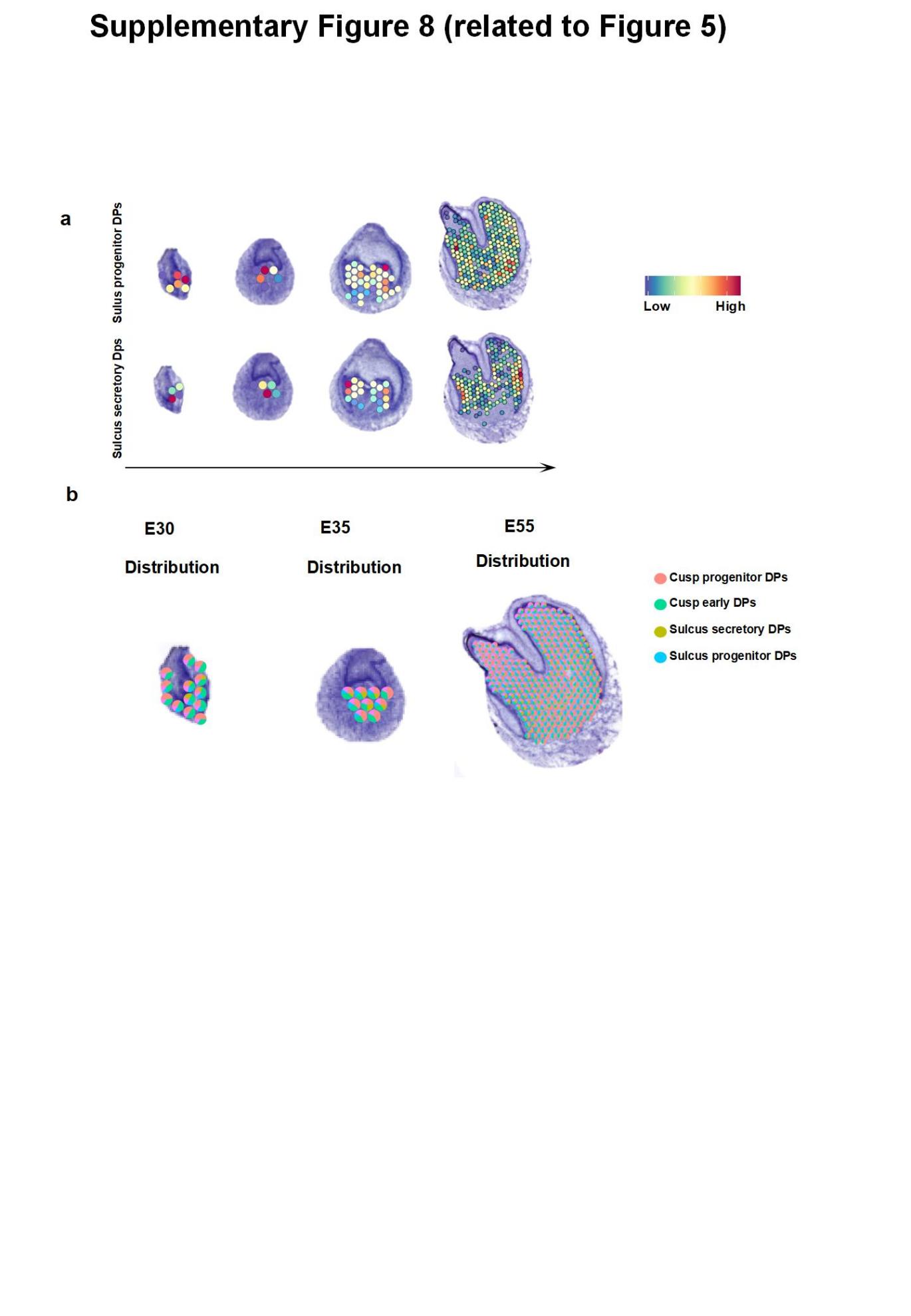


Figure S10. Development of papilla clusters, related to Figure 5

(**a**-**b**), Conjoint analysis of single cell and spatial transcriptome sequencing showing the spatial distributions of sulcus progenitor DPs and sulcus secretory early DPs at the bud stage, cap stage, bell stage and the differentiation stage.


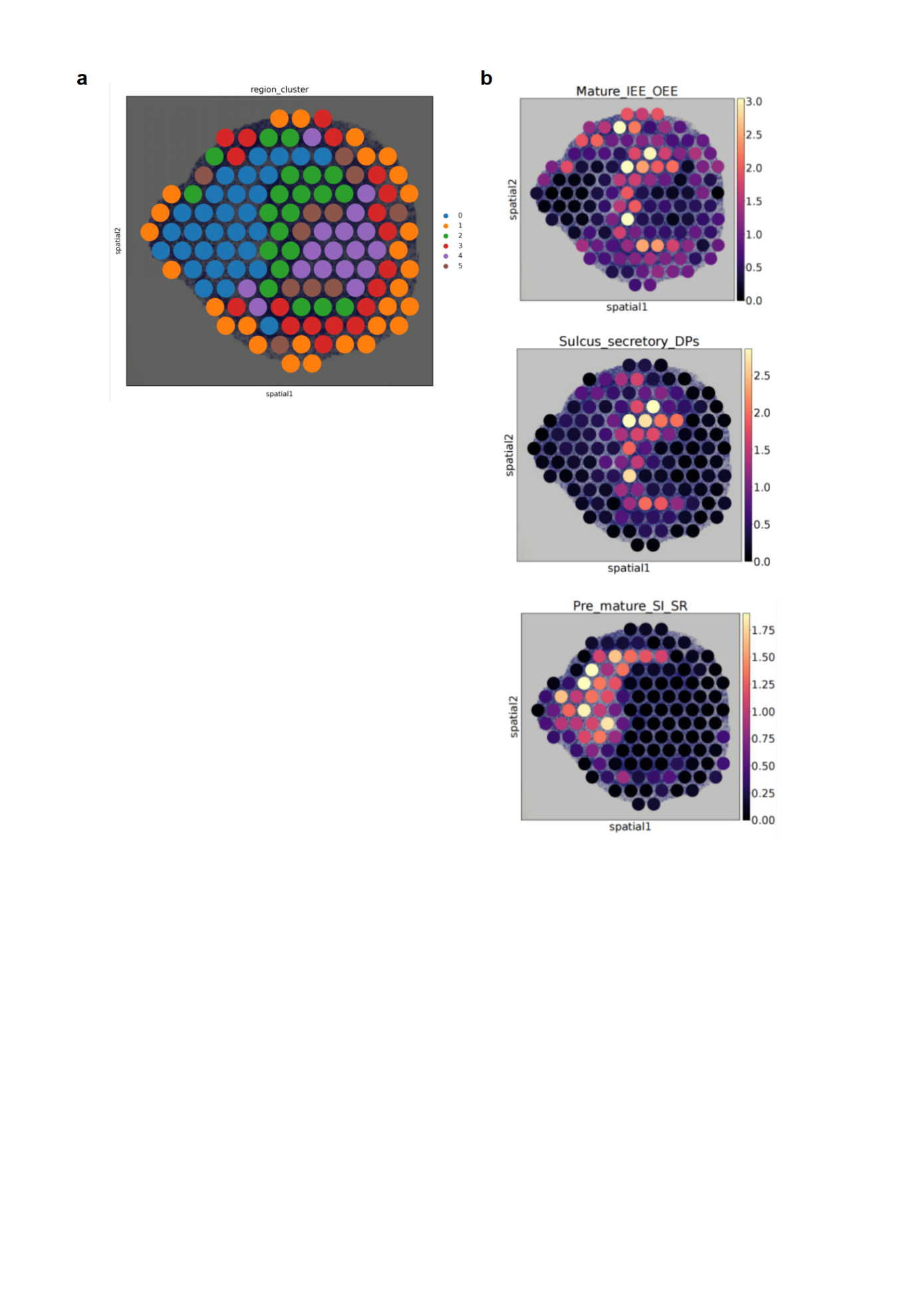


Figure S11. Celltype abundance on spatial.

**a** Spatial map of the clustering of cell type components. **b** mature_IEE_OEE, Sulcus_secretory DPs and abundance distribution of Pre_mature_SI_SR cell subtype.


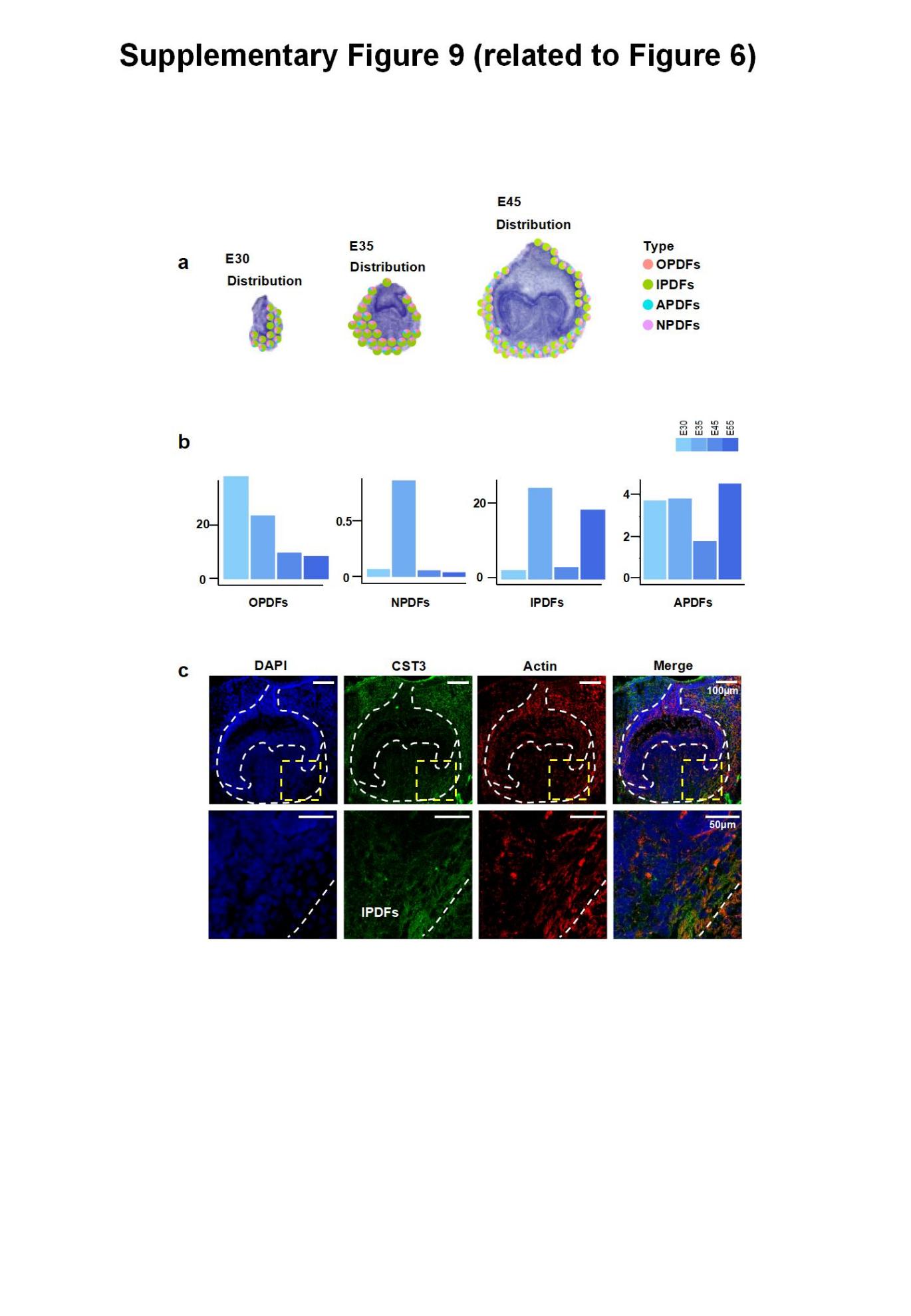


Figure S12. Development of papilla follicles, related to Figure 6

**a** Conjoint analysis of single cell and spatial transcriptome sequencing showing the spatial distributions of DFs at the bud stage, cap stage, bell stage and the differentiation stage. **b** Bar plots showing the time distribution characteristics of dental follicle populations, indicating the occurrence sequence of progenitor clusters and differentiated clusters. **c** Immunofluorescence staining visualizing the main expression of CST3 in IPDFs cells.


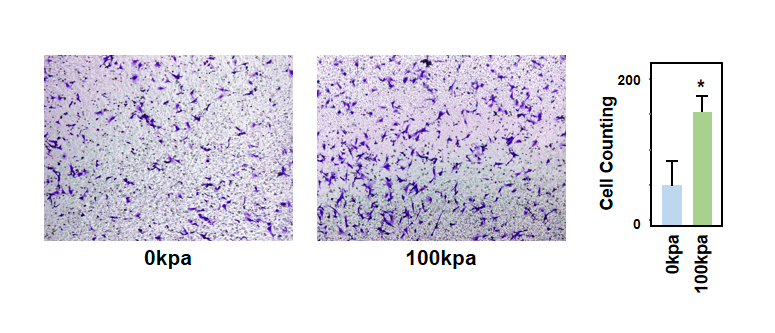


Figure S13. Validation of the migratory function of ameloblast-lineage cells under stress *in vitro.*, related to Figure 7

The migratory capacity of ameloblast-lineage cells was significantly enhanced under a compressive stress of 100kpa.*P < 0.05; two-tailed unpaired Student’s t-test.
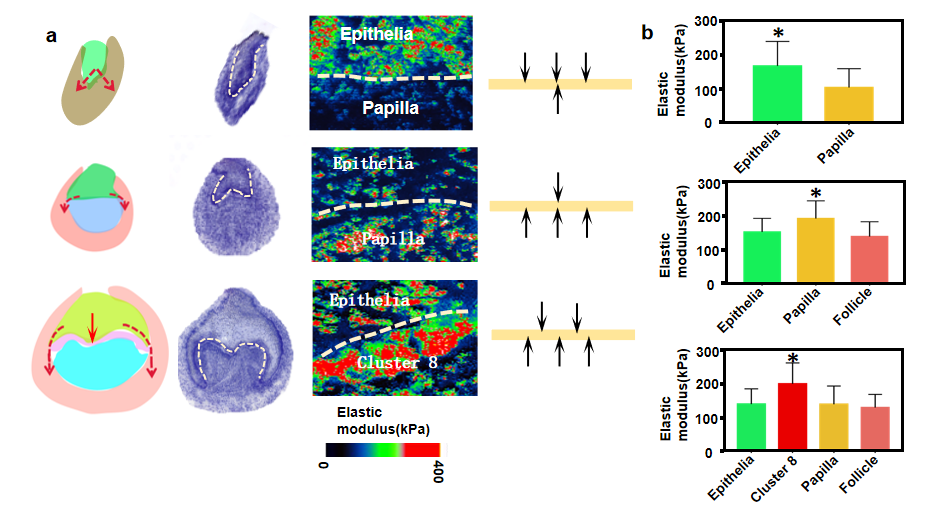


Figure S14. The clusters stiffness in ST were confirmed by AFM, related to Figure 7

**a** Modeling cell migration and thermal mapping of cell stiffness at the interface between tooth germ epithelium and mesenchyme. **b** Quantification of tissue stiffness showing that the cluster 8 were the stiffest at the epithelium-mesenchyme interface, as measured by AFM. *P < 0.05; two-tailed unpaired Student’s t-test.


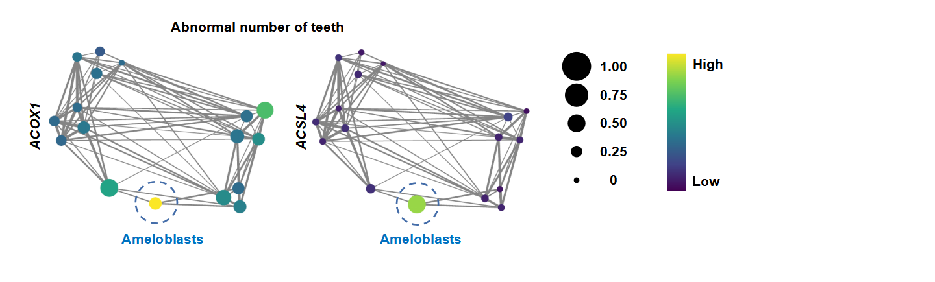


Figure S15.Cell source analysis of the selected genes related to dental dysplasia, related to Figure 8. Graph abstraction overlay visualizing the cluster expression of genes associated with abnormal number of teeth (*ACOX1* & *ACSL4*).
